# Preservation of Tetherin and CD4 Counter-Activities in Circulating *Vpu* Alleles despite Extensive Sequence Variation within HIV-1 Infected Individuals

**DOI:** 10.1371/journal.ppat.1003895

**Published:** 2014-01-23

**Authors:** Suzanne Pickering, Stephane Hué, Eun-Young Kim, Susheel Reddy, Steven M. Wolinsky, Stuart J. D. Neil

**Affiliations:** 1 Department of Infectious Disease, King's College School of Medicine, Guy's Hospital, London, United Kingdom; 2 MRC Centre for Medical Molecular Virology, University College London, London, United Kingdom; 3 Department of Medicine, Northwestern University, Chicago, Illinois, United States of America; University of Massachusetts Medical School, United States of America

## Abstract

The HIV-1 Vpu protein is expressed from a bi-cistronic message late in the viral life cycle. It functions during viral assembly to maximise infectious virus release by targeting CD4 for proteosomal degradation and counteracting the antiviral protein tetherin (BST2/CD317). Single genome analysis of *vpu* repertoires throughout infection in 14 individuals infected with HIV-1 clade B revealed extensive amino acid diversity of the Vpu protein. For the most part, this variation in Vpu increases over the course of infection and is associated with predicted epitopes of the individual's MHC class I haplotype, suggesting CD8+ T cell pressure is the major driver of Vpu sequence diversity within the host. Despite this variability, the Vpu functions of targeting CD4 and counteracting both physical virus restriction and NF-κB activation by tetherin are rigorously maintained throughout HIV-1 infection. Only a minority of circulating alleles bear lesions in either of these activities at any given time, suggesting functional Vpu mutants are heavily selected against even at later stages of infection. Comparison of Vpu proteins defective for one or several functions reveals novel determinants of CD4 downregulation, counteraction of tetherin restriction, and inhibition of NF-κB signalling. These data affirm the importance of Vpu functions for *in vivo* persistence of HIV-1 within infected individuals, not simply for transmission, and highlight its potential as a target for antiviral therapy.

## Author Summary

The accessory protein Vpu, encoded by HIV-1, performs at least two major roles in the virus life cycle, namely the degradation of newly synthesized CD4 molecules and the counteraction of a host antiviral protein, tetherin. These activities promote the release of infectious viruses from host cells, and recent evidence suggests that Vpu function has been crucial for the cross-species transmission of HIV-1 from chimpanzees, and its subsequent pandemic spread in humans. Here we studied the functional variation in Vpu in infected individuals. We found that the Vpu amino acid sequence can be highly variable within an individual, and that this variation is likely to result from host immune responses targeting antigens derived from Vpu. However, despite this variation, Vpu's major functions are preserved, with only a minority of circulating alleles showing defects throughout the course of infection. These data suggest that defective Vpu proteins are selected against within the infected individual, implying that Vpu functions are critical for HIV-1 replication throughout natural infection, not simply at transmission. Therefore Vpu may represent a novel target for antiviral therapy to augment current treatment strategies for HIV/AIDS.

## Introduction

The HIV-1 genes *nef*, *vpu*, *vif* and *vpr* are known as accessory genes and early *in vitro* studies showed them dispensable for viral replication in some tissue culture cell lines [1]. *In vivo*, however, these proteins are essential for the transmission and persistence of immunodeficiency viruses. Vpu, in particular, is thought to have been pivotal to the ability of HIV-1 group M to establish pandemic infection in humans following transmission from chimpanzees [2], [3]. Expressed late in the viral life cycle, it functions during viral assembly to facilitate efficient egress of infectious viral particles, through the degradation of CD4 in the endoplasmic reticulum (ER) and the counteraction of the interferon-induced antiviral protein tetherin (BST2/CD317) [4], [5]. By antagonising tetherin, Vpu also acts to evade innate immune sensing of budding viral particles by repressing pro-inflammatory signalling events triggered by tetherin [6]–[8]. In recent years, Vpu has been implicated in other immunomodulatory functions, such as the downregulation of NTB-A/SLAMF6 [9] and poliovirus receptor (PVR/CD155) [10] to evade NK cell recognition of HIV-1 infected cells, and the removal of CD1d from the surface of dendritic cells, inhibiting lipid antigen presentation to NK-T cells [11]. Furthermore, signature residues in the C-terminus of Vpu are associated with NK cell escape in KIR2DL2 positive individuals [12].

Vpu is found only in the SIVcpz/HIV-1 lineage of primate lentiviruses, yet its ability to counteract tetherin and downregulate CD4 is inconsistent throughout the members of this family. Vpu proteins from HIV-1 group M tested to date can perform both functions; the majority of available group N Vpu proteins weakly counteract tetherin and do not degrade CD4, although show signs of adapting to human tetherin [13]; while in contrast, group O and P proteins can degrade CD4 but are fundamentally ineffective at counteracting tetherin [3], [14]–[16]. The Vpu of the precursor virus, SIVcpz, can degrade CD4 but is ineffective against both chimpanzee and human tetherins; in infected chimpanzees, Nef performs this role by targeting a region of chimpanzee tetherin deleted in its human orthologue [3], [17]–[19]. Vpu is absent from the genome of HIV-2, therefore the envelope protein has adapted to the role of tetherin antagonist in these viruses [20], whilst Nef alone downregulates CD4. Thus, of all the immunodeficiency viruses able to infect humans, HIV-1 group M is the only virus group able to both degrade CD4 in the ER and counteract and ultimately degrade tetherin, suggesting that the Vpu protein may play a key role in the transmissibility and pathogenicity of this group, and potentially its pandemic status [3]. Most characterisation of group M Vpu thus far conducted has been of the prototypical molecular clone virus, NL4.3 (reviewed in [4]), on panels of representative Vpus from different clades [3], [14], [15], [21], or on bulk-cloned proviral sequences [22] necessitating an in depth study of natural *vpu* alleles.

In mice, tetherin activity moderates the replication and pathogenicity of murine retroviruses [23]–[25], suggesting it plays an antiviral role *in vivo*. Overcoming the physical block to virus release is one obvious reason that Vpu tetherin antagonism might be essential for HIV-1 *in vivo*. However, whether tetherin can block cell-to-cell transmission of HIV-1, likely to be the predominant mode of systemic viral spread in lymphoid tissue, is controversial and cell-type dependent [26]–[28]. The presence of tetherin at the virological synapse can, in some circumstances, enhance cell-to-cell virus spread [27], in agreement with early observations that Vpu-deleted viruses spread faster in tissue culture [29]. Moreover, in all studies directly addressing the role of tetherin in cell-to-cell spread of HIV-1 the effects, either positive or negative, have been weak. Alternatively, downstream consequences of tetherin restriction *in vivo*, in particular the recently described pattern recognition activity of tetherin [6]–[8], may put extra selective pressure on the maintenance of Vpu function throughout infection. Tetherin expression is upregulated on HIV-1 target cells in infected individuals [30]. Interestingly, sequence changes in Vpu have been documented in patients co-infected with hepatitis C virus after treatment with pegylated type-1 interferon [31]. This prompted us to question whether tetherin antagonism is important throughout HIV-1 infection *in vivo*, or whether functional variability in this attribute is tolerated after the virus has established a systemic infection.

Using single genome amplification of *vpu* alleles from infected individuals and optimised assays for the three major functions of Vpu, we completed a comprehensive study of Vpu function in natural HIV-1 infection. Single genome amplification eliminates sample bias and PCR-based recombination and provides a representation of the proportion of viral alleles circulating at one time point, whilst allowing direct progression to tractable functional assays. In the latter feature, at present, it has an advantage over deep/next generation sequencing approaches. Furthermore, deriving *vpu* sequences from virions rather than cell-derived provirus is entirely representative of one particular timepoint, and less likely to contain defective variants in comparison. The aims of the study were twofold: to comprehensively characterise Vpu sequence variation, immune pressure and major functions from natural infection; and to inform current structure-function studies of Vpu by investigating naturally defective and sub-optimal Vpu proteins.

## Results

### *Vpu* sequence repertoire and variation in infected individuals

**Figure 1:**
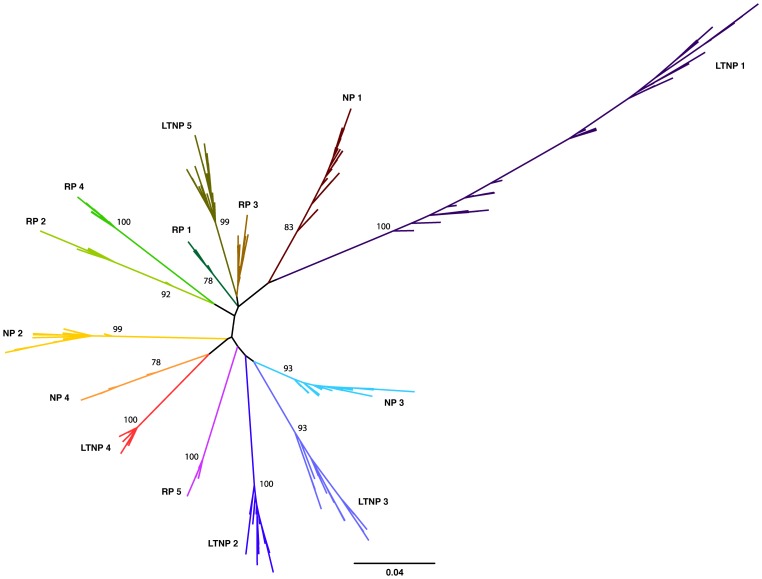
*In vivo* variation of HIV-1 clade B *vpu.* Unrooted maximum likelihood phylogeny of 851 *vpu* nucleotide sequences derived from 25 samples from 14 HIV-1-infected individuals. Individuals were classified according to time from seroconversion to progression to AIDS: 5 rapid progressors (RPs), 4 normal progressors (NPs) and 5 long-term non-progressors (LTNPs) with sequences from each individual coloured and labelled accordingly. Bootstrap supports (% confidence) are shown at the base of the branch for each individual. Branch lengths indicate the number of nucleotide substitutions per site.

Vpu sequences were derived from actively replicating plasma virus from 14 HIV-1-infected individuals: 5 long-term non-progressors (LTNP), 5 rapid progressors (RP), and 4 normal progressors (NP), detailed in **Table 1** [32]–[34]. Patients were classified as follows, according to standard MACS criteria: individuals that progressed from seroconversion to AIDS in less than 5 years were designated RPs; 5–10 years NPs; and greater than 10 years for LTNPs. All individuals were treatment naïve, both during and prior to the time of sampling. Up to three different time points were obtained from each individual, ranging from seroconversion (0 years) to 10.4 years, with 1–2 year and 3–4 year time points obtained for each individual where possible. To represent fully the *vpu* repertoire in each plasma sample and to maximise the probability of isolating representative minor viral variants, at least 29 sequences were obtained from each sample, yielding a total of 851 *vpu* sequences from all 25 plasma samples (**Table 1**). All 851 nucleotide sequences obtained were aligned and assembled into a maximum likelihood phylogenetic tree (**Figure 1**), with *vpu* sequences from each infected individual forming a monophyletic group, in accordance with their distinct origin. Sequences from individuals with different progression rates to disease did not cluster in proximity to each other, indicating the lack a direct relationship between a specific *vpu* sequence and pathogenic outcome. Individual phylogenetic trees of *vpu* sequences from each of the 14 individuals are shown in **Figure S1**.

**Table 1:** Study subjects, sequence details and natural *vpu* variation.

Subject	Time (yrs)^1^	Viral load^2^	CD4 counts (per ml)	Sequence number^3^	Unique sequences^4^		Genetic distance (substitution/site)		
					NT	AA	NT	AA	
							Mean	Mean	Max
RP 1	1.2	32,280	186	36	18	14	0.007	0.011	0.051
RP 2	1.0	106,070	442	35	22	14	0.032	0.020	0.051
RP 3	2.0	109,736	166	36	28	22	0.018	0.033	0.080
RP 4	2.0	40,403	147	33	21	13	0.013	0.023	0.079
RP 5	1.1	7,095	770	34	13	10	0.007	0.019	0.065
NP 1	1.6	63,265	636	33	17	9	0.010	0.016	0.058
	3.5	27,610	498	43	21	13	0.015	0.027	0.078
NP 2	2.0	16,221	513	34	19	14	0.010	0.022	0.063
	4.2	62,022	370	33	25	18	0.019	0.029	0.077
NP 3	0	52,367	750	41	12	6	0.005	0.004	0.044
	1.6	55,603	553	38	14	10	0.006	0.015	0.060
	3.9	266,082	127	33	18	12	0.017	0.030	0.077
NP 4	4.9	303,776	309	30	14	8	0.009	0.015	0.051
LTNP 1	1.1	28,653	429	32	20	18	0.022	0.058	0.168
	4.4	106,333	758	32	18	13	0.026	0.060	0.179
	10.4	82,071	374	41	27	14	0.039	0.073	0.224
LTNP 2	1.2	8,096	672	31	17	7	0.012	0.008	0.067
	3.6	31,766	864	35	21	8	0.020	0.019	0.064
LTNP 3	1.2	6,997	425	29	12	10	0.013	0.035	0.135
	4.5	29,229	428	30	18	15	0.029	0.058	0.135
LTNP 4	0	448,694	596	31	4	2	0.001	0.001	0.012
	1.1	10,268	276	31	9	5	0.003	0.004	0.034
	3.5	12,250	296	33	15	7	0.007	0.008	0.051
LTNP 5	1.1	54,053	986	36	29	20	0.017	0.043	0.181
	3.6	30,343	504	31	24	22	0.020	0.045	0.107

^1^ The time point of the plasma sample, in years post-seroconversion.

^2^ Viral RNA copies/ml peripheral blood.

^3^ The total number of *vpu* sequences obtained per sample by single genome amplification.

^4^ Unique sequences represent the number of alleles per sample after stripping of duplicates.

The mean intra-patient nucleotide and amino acid diversity for each individual *vpu* repertoire is shown in **Table 1**. *vpu* sequence diversity did not correlate with disease progression rates, with individuals within the groups harbouring a range of sequence diversity levels (LTNP 1 and 4, for example). As expected, mean intra-patient nucleotide and amino acid diversity increased over time in all individuals. There was no correlation between genetic distance and the number of sequences obtained per sample, suggesting that the viral repertoire in each sample had been fully represented (**Figure S2**). Vpus from LTNP 1 showed the highest level of genetic diversity, as evident from **Figure 1** and **Table 1**, however, it should be noted that an extra 10.4 year time point was analysed from this individual; sequence diversity was comparable to others from the same progression group at equivalent time points. We found no indication of APOBEC3-mediated changes acting on the individual *vpu* populations (data not shown).

### Functional Vpu repertoires from infected individuals

**Figure 2:**
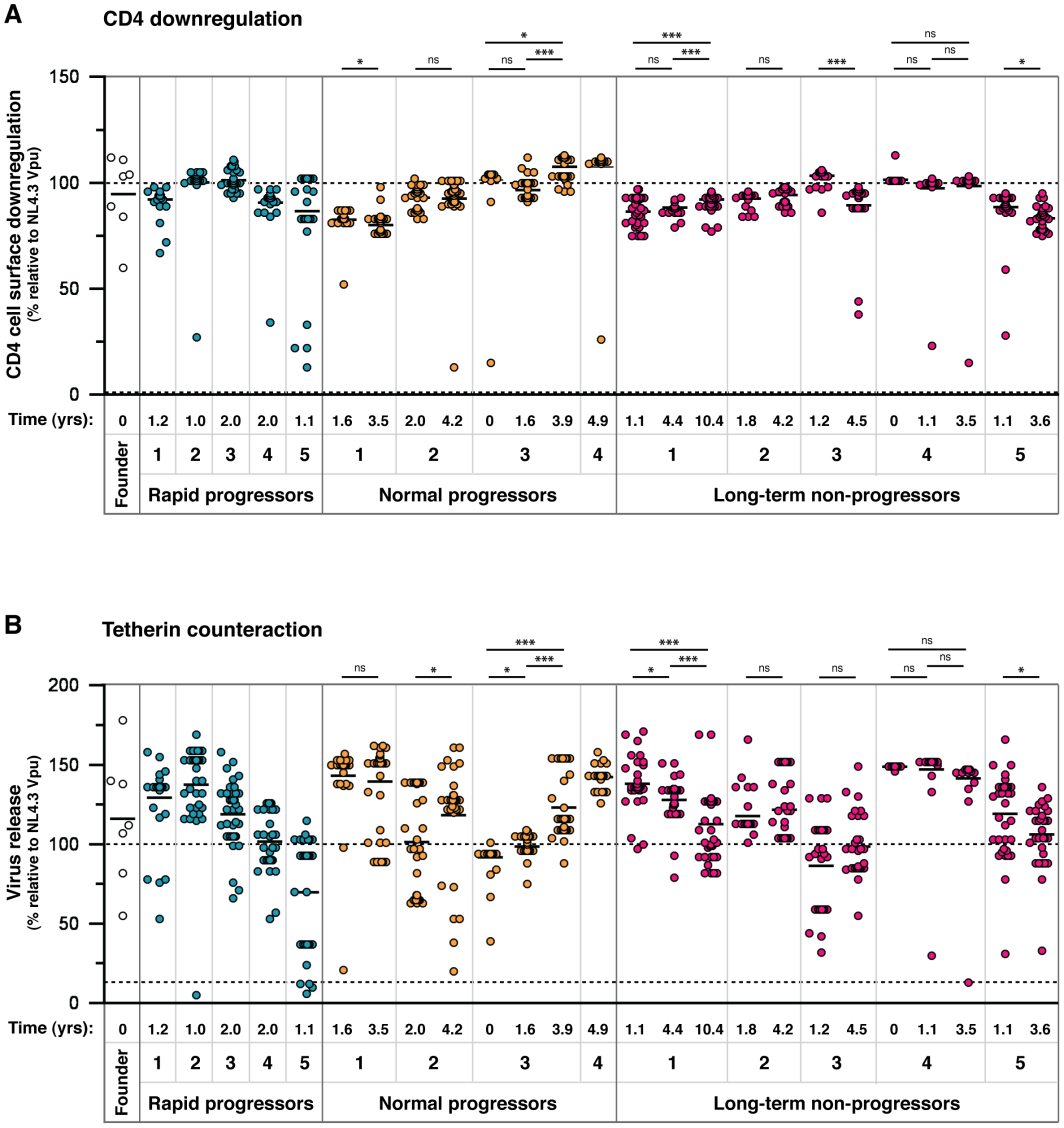
Analysis of CD4 downregulation and tetherin antagonism of 304 natural Vpu alleles. Representatives of all amino acid sequences obtained through SGA were cloned and tested in standardised assays for the two major functions of Vpu: (**A**) cell surface CD4 downregulation and (**B**) tetherin counteraction. The time point of each sample, in years post-seroconversion, is indicated beneath the graph with the patient identification and progression group. The function of each Vpu is represented relative to the prototypical Vpu from NL4.3, as indicated by a dashed line at 100%, and the functions of all other Vpus are represented as a percentage thereof. The NL4.3 S52,56A Vpu mutant, defective for both CD4 downregulation and tetherin counteraction activity, is indicated by a dashed line (at 0% on the CD4 downregulation graph and 13% on the tetherin counteraction graph). Each symbol represents the average of a minimum of three independent experiments, weighted to represent the number of sequences obtained per sample with that particular amino acid sequence (allele frequency), to give an overall proportional representation of function per time point. Means for overall Vpu function for each time point are shown as short horizontal lines. Significant differences between time points from each individual are indicated by asterisks. Briefly, the assays were performed as follows: (**A**) HeLa-TZMbl cells were co-transfected with 100 ng of pCRVI-Vpu or empty vector (EV) plasmid and 100 ng of pCR3.1-eGFP. 24 hours later cell surface CD4 levels were analysed by flow cytometry. CD4 downregulation was determined by comparing median fluorescent intensities of CD4 in the presence and absence of Vpu, with the downregulation achieved by NL4.3 Vpu set at 100%. Note that the absolute value of CD4 reduction achieved by NL4.3 was 80%±6. (**B**) 293T cells were transfected with a fixed dose (50 ng) of pCR3.1-hu-tetherin in combination with 500 ng of NL4.3-del Vpu proviral plasmid and 25 ng of pCRVI-Vpu. 48 hours later the supernatants are removed from the cells and assayed on Hela-TZMbl cells for the quantity of infectious virus. The 100% line represents the amount of infectious virus released in the presence of NL4.3, to allow direct comparisons between the CD4 downregulation and tetherin counteraction assays. 25 ng of pCRVI-Vpu was used as this quantity produced the same amount of Vpu protein as that of the full-length NL4.3 molecular clone, as determined by Western blot analysis.

Of the 851 *vpu* sequences obtained, 456 had unique nucleotide sequences, and 304 unique amino acid sequences. Of these 304 alleles, five contained readily detectable mutations (i.e. deletions or frame-shifts), specifically: two contained a premature stop codon (LTNP2v14_4_51 and LTNP5v22_5_71, resulting in a 6- and 1-amino acid C-terminal truncation, respectively), one contained a frame-shift (RP2v16_1_5), and two contained a 1-amino acid N-terminal deletion (LTNP1v11_4_3 and 5_38). The other 299 Vpus were 81 amino acids in length and thus potentially functional. Since a single amino acid change can impact on the function of a protein, all 304 Vpu alleles were cloned and tested in standard functional assays for CD4 downregulation and tetherin counteraction. Samples were weighted according to how many genomes were isolated with a particular amino acid sequence, thus representing the proportions of functional and non-functional Vpus present in a given sample (**Figure 2**). Vpu from the HIV-1 clade B molecular clone NL4.3 was used as the prototypical Vpu in all assays, to which the functions of patient-derived Vpu proteins were compared. Mutant Vpus derived from NL4.3 with defects in tetherin counteraction (A14L), or both tetherin counteraction and CD4 downregulation (S52,56A and AW14,22LA) were included in each assay as negative controls. We also tested Vpus derived from a panel of HIV-1 clade B transmitted/founder viruses as representatives of earliest available replicating virus [35]. Assay cut-offs were determined by the performance of the entire Vpu population, with the threshold for sub-optimal or defective activity set at one standard deviation below the mean: for CD4 downregulation this was 73.7 (mean Vpu function 90.3%, standard deviation 16.6); for tetherin counteraction this was 81 (mean Vpu function 114%, standard deviation 33). Based on these criteria, 17 Vpus were suboptimal/defective for CD4 downregulation, and 41 for tetherin counteraction.

Interestingly, the founder virus Vpus displayed a spread of function representative of the 304 patient-derived alleles, with CH040 Vpu showing sub-optimal activity. There were no discernible correlations between either of the two Vpu functions and disease progression groups or time post-seroconversion. Neither could we detect a correlation between tetherin counteractivity and viral load; although in six of the eight individuals with more than one time point, anti-tetherin function did increase with an increase in viral load (data not shown), but changes were not significant. CD4 downregulation activity was highly maintained across all individuals and time points. Vpus from the same individual had a narrow range of function, with defective Vpus set apart from the rest of the group, indicative of an intrinsic activity of each Vpu population. The spread of tetherin counteraction was broader than that of CD4 downregulation, perhaps due to a more complex mechanism and more regions of the protein involved in tetherin downregulation and degradation. In individuals in which there was a discernable group of suboptimal Vpus, the group was diminished in number at the later time point (NP 2, NP 3, LTNP 3), suggesting ongoing pressure for Vpu to maintain optimal function throughout infection.

To assess the impact of single, and thus potentially transitory, variants, the functional data from **Figure 2** were re-plotted after the removal of all Vpus represented by one single genome, showing only the variants represented by multiple genomes (**Figure S3**). For the most part, the data remain unaffected, with only the degree of significance changing over time in some individuals. Specifically, for CD4 downregulation the decreases seen for NP 1 and LTNP 5 had higher *p* values when single variants were removed, and the NP 2 increase became significant (*p* = 0.042). For tetherin counteraction, the increase seen for NP 2 had a higher *p* value, as did the decreases seen for LTNP 1 and LTNP 5. For some individuals, removal of single variants lead to all values for one time point being identical, and in these cases statistical analyses could not be performed (LTNP 3, LTNP 4).

### NL4.3 Vpu – A suboptimal tetherin antagonist?

**Figure 3:**
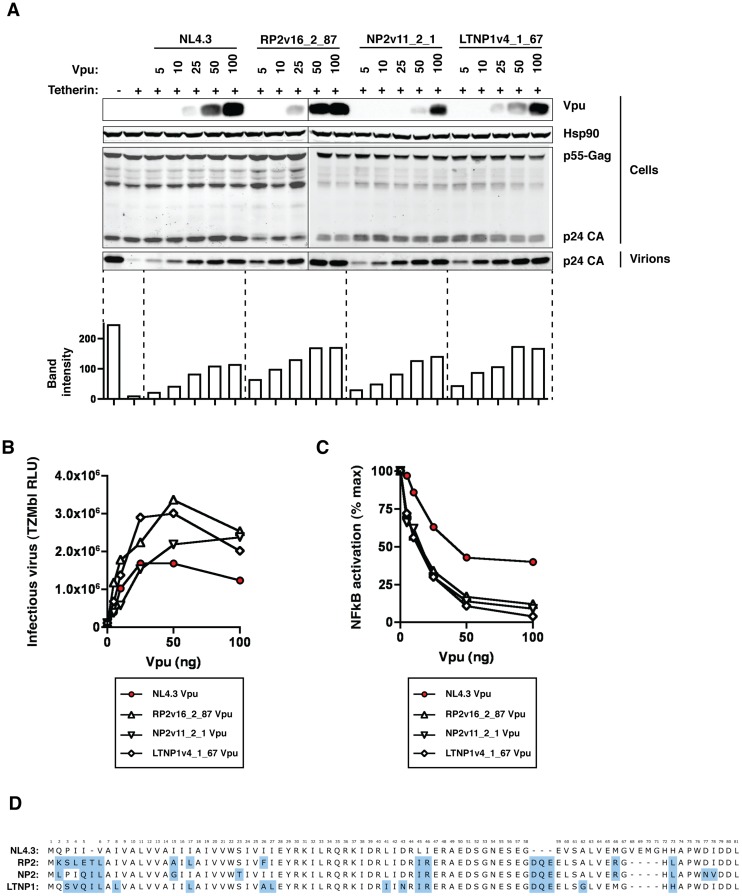
NL4.3 Vpu as a sub-optimal tetherin antagonist. NL4.3 Vpu and three highly active patient-derived Vpus, RP2v16_2_87, NP2v11_2_1 and LTNP1v4_1_67, were tested at a range of concentrations for their ability to counteract tetherin in a standard virus release assay. 293T cells were transfected with NL4.3 wild-type (HIV-1 wt) or NL4.3 del Vpu (HIV-1 ΔVpu), together with 50 ng human tetherin and 0, 5, 10, 25, 50 and 100 ng of each pCRVI-Vpu. (A) Cell supernatant was filtered and sucrose purified to yield cell-free virions. Cell lysates were analysed for Vpu, Hsp90 (loading control) and p24 protein expression, and the p24 protein levels in the pelleted virions quantified as a representation of virus released from the cells. (B) The quantity of infectious virus released into the cell supernatant in the presence of 0, 5, 10, 25, 50 and 100 ng of each of the Vpu proteins was assayed on HeLa-TZMbl cells. Error bars represent standard deviation from 3 independent experiments. (C) Inhibition of transient tetherin-mediated NF-κB activation by Vpu. Fold activation of a luciferase NF-κB reporter gene by expression of 50 ng human tetherin is calculated relative to a GFP control in the presence of increasing concentrations of NL4.3, RP2v16_2_87, NP2v11_2_1, and LTNP1v4_1_67 Vpu, and results are presented relative to the mean signal obtained in the absence of Vpu (% max) (D) Amino acid alignment of NL4.3 RP2v16_2_87, NP2v11_2_1, and LTNP1v4_1_67 Vpus.

The majority of our natural Vpu proteins had tetherin counter-activity superior to that of the NL4.3 Vpu prototype. The ability of NL4.3 to down-regulate CD4, however, appeared near-optimal compared to primary Vpu proteins. Direct comparisons between NL4.3 Vpu and three representative patient-derived Vpus from each of the three progression groups (RP2v16_2_87, NP2v11_2_1, and LTNP1v4_1_67), confirmed that NL4.3 performed notably poorer than typical clade B Vpus in tetherin counteraction (**Figure 3**). The three natural Vpus differed in sequence by 7 to 10 amino acids from the sequence of the Consensus B Vpu obtained from the Los Alamos National Laboratory HIV database. The expression of the patient-derived Vpus was not greater than that of NL4.3 by Western blot (**Figure 3**), and in titration experiments, up to 100% more infectious virus is released in the presence of the patient-derived Vpu compared to NL4.3 Vpu. The ability to downregulate CD4 was optimal for the four proteins (RP2v16_2_87 bearing 101% activity relative to NL4.3, NP2v11_2_1 89%, and LTNP1v4_1_67 86%), supporting the notion that NL4.3 is inferior to natural Vpu proteins only in tetherin counteraction. The superiority of the patient-derived Vpus was further demonstrated by their suppression of tetherin-mediated NF-κB activation. In transient tetherin signalling assays, in which tetherin is overexpressed to mimic receptor clustering and activate NF-κB [7], various Vpu constructs were titrated and assessed for their ability to reduce activation of an NF-κB reporter construct by tetherin. At 25 ng of Vpu the residual tetherin signalling activity in the presence of RP2v16_2_87, NP2v11_2_1 and LTNP1v4_1_67 was 34, 30 and 30% respectively, compared to 63% in the presence of NL4.3. All patient-derived Vpus almost completely abolished tetherin signalling at the higher Vpu expression level of 100 ng. Comparison of the NL4.3 and patient-derived Vpu amino acid sequences highlights differences in the C-terminal portion of the cytoplasmic tail, notably in the 2^nd^ alpha-helix where putative trafficking domains and acidic patches are positioned differently relative to the conserved phosphorylated serines (**Figure 3**).

### Structure-function analyses of *vpu* alleles: CD4 vs tetherin counteraction

**Figure 4:**
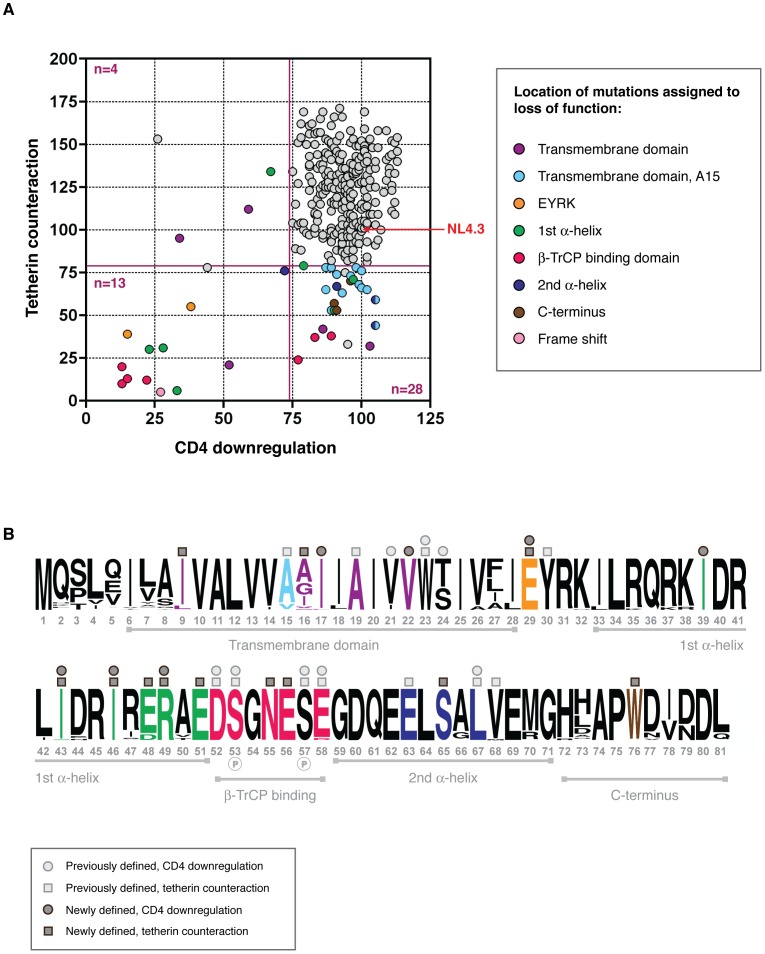
Structure-function analysis of sub-optimal and non-functional Vpus. (A) Functional profiles for the 304 tested Vpus, shown as tetherin counteraction vs CD4 downregulation, with both functions measured as a percent of NL4.3 Vpu activity (100%, indicated by red arrow; see **Figure 2** for details). Non-functional and sub-optimal Vpus were defined as 0–73.7% of NL4.3 Vpu function for CD4 downregulation, and 0–81% for tetherin counteraction, determined by one standard deviation below the mean activity of all Vpus tested. Cutoffs are indicated by dark red solid lines. Vpus were categorised according to whether they were defective for tetherin counteraction (n = 28), CD4 downregulation (n = 4), or both (n = 13), then compared to their closest functional relatives and to the Vpu population as a whole, to pinpoint the amino acid changes responsible for the defect. Each defective/sub-optimal Vpu is coloured according to the location of the inactivating mutation, as detailed in the key, and then highlighted in (B). (**B**) A logo plot of the 304 natural Vpu proteins, illustrating the variation at each amino acid position at the population level and annotated to show the major domains of the Vpu protein. Amino acids with previously known contributions to tetherin counteraction and/or CD4 downregulation are indicated by light grey squares and/or circles above the relevant position, respectively. Sites attributed to loss of function in this study are coloured according to (A) and indicated by dark grey squares and/or circles for tetherin counteraction and/or CD4 downregulation. Only the position, rather than the amino acid identity, is shown; for the specific mutations see **Figure S4**. Note that an unannotated residue does not necessarily mean it is not essential to either or both of the functions, but rather that this site was invariant. See **Figure S4** for further details, and **Table S1** for the complete database of Vpu sequences and functions.

When compared for CD4 downregulation and tetherin counteraction activity, each Vpu had a unique functional profile, as shown in **Figure 4**. The vast majority of Vpus were able to perform both functions (n = 263; 86.5%), yet there were sufficient numbers of defective proteins to merit investigation of structure-function relationships. Vpus with levels of activity ranging from defective to sub-optimal (defined as 0–81% of NL43 activity for virus release; 0–73.7% for CD4 downregulation) were categorised according to whether they had defects in tetherin counteraction only (n = 23; 7.6% of all Vpus), CD4 downregulation only (n = 7; 2.3%), or both (n = 11; 3.6%). Of note, there were more Vpus defective for tetherin counteraction alone than there were for CD4 downregulation only, and the overall range of function for tetherin counteraction was broader than that of CD4 downregulation.

Comparing Vpu sequences from different viral isolates in order to identify regions of functional interest can often be problematic due to multiple differences between given sequences [14], [21]. However, the advantage of using sequences obtained by SGA is that, in the majority of cases, each defective or suboptimal Vpu has a functional relative that differs by only one or two amino acids. Thus, by comparing the sequences of defective Vpus to their closest functional relatives, and then to the entire Vpu repertoire, in most cases the amino acid change or changes responsible for the defect could be identified (**Figure 4**, **Figure S4** and **Table S1**). Proteins defective for both CD4 downregulation and tetherin counteraction (i.e. less than the 81% cutoff for tetherin and 73.7% for CD4) contained a frame-shift (n = 1), an A19E change in the transmembrane domain (n = 1), mutations of the highly conserved regions just prior to (E29K; n = 2) and within (II43,46SL, R49G, R49T; n = 3) the first alpha-helix, and in the DSGNES hinge region between the two cytoplasmic alpha helices (D52V, SN53,55RH, S53N, E58K; n = 4), which contains two phosphorylated serines essential for interactions with the E3 ubiquitin ligase complex SCF^β-TrCP^, central to Vpu's function. Since the expression of Vpus with defects in both functions could not be assumed, expression of these proteins was verified by Western blot analysis, and although variable, all but one Vpu could be detected. The latter, when compared with known functional proteins from the same sample, was from a population of Vpus not recognised by the anti-Vpu antibody used for Western blot analysis (**Figure S4**).

### CD4-specific mutations

Since there were only four Vpus with a defect in CD4 downregulation alone, this presented fewer opportunities for determining regions specific only to this function. Indeed, in contrast to tetherin counteraction, there is little consensus in the literature regarding individual amino acids or motifs in Vpu specifically governing CD4 downregulation. However, of the three in which specific changes could be assigned to loss of function, these mapped to conserved residues in the first alpha helix of the Vpu cytoplasmic tail (n = 1) and transmembrane region (n = 2), specifically I17T, V22A and I39L.

One caveat to the CD4-only defects is that, while the tetherin functions for all of them were more than 81% that of NL4.3, in many cases they were still suboptimal compared to the better performing Vpus in the data set. Of note, the transmembrane residues assigned to CD4 downregulation defects were highly conserved.

### Tetherin counteraction-specific mutations

Tetherin-specific functional mutations were tracked to the transmembrane domain (n = 14), to conserved residues in the first alpha-helix (E48, n = 2), to the conserved DSGNES hinge region (n = 3); to the ExxxLV motif (and flanking residues) in the second alpha-helix (n = 3); and to a conserved tryptophan in the Vpu C terminus (n = 3); with 2 Vpus with unassignable defects (**Figure 4** **, Figure S4**). At least two regions in Vpu have previously been assigned specific functions in the context of tetherin counteraction: in the transmembrane domain, alanines at position 15, 19 and a tryptophan at position 23 (positions 14, 18 and 22 in NL4.3 for reference), aligned along one face of the transmembrane helix, form an interacting surface with the tetherin transmembrane region [36]; in the second alpha helix of the cytoplasmic domain, an ExxLV motif, a putative sorting signal, plays a role in trafficking and degradation of Vpu/tetherin complexes [37]. A high proportion of the mutations that affected only tetherin counteraction mapped to an A15 change to a valine or threonine, (n = 14), and for the most part resulted in a modest reduction in tetherin counteraction. Since 12.2% (n = 37) of all Vpus contained a valine at this position, and a further 1.6% (n = 5) a threonine, and not always immediately conferring a disadvantage in comparison with NL4.3, the effect at this position is clearly dependent on context and may potentially weaken the interaction with tetherin. However, when comparing V15 and T15 Vpus with matched Vpus from the same infected individual with alanines at this position (when available), rather than with NL4.3 Vpu, all demonstrated at least a 50% relative defect in virus release (**Table S1**). Interestingly, in two individuals, NP 2 and LTNP 3, V15 or T15 Vpus make up a large population of sub-optimal Vpus (35.3 and 100% of the 1–2 year time point respectively), with the overall function of these time points falling at or below the level of NL4.3. In both cases these are significantly fewer in proportion in the following time point (using Fisher's exact test: NP 2 *p* = 0.043; LTNP 3 *p* = 0.0046), indicative of them being selected against, and the overall function of the subsequent time point is significantly higher (**Figure 2**; NP 2, LTNP 3).

In contrast to the variation seen at position 15, only one Vpu contained a mutation at position 19 (NP1v5_1_80, A19E), leading to a loss of anti-tetherin function and a severe (2-fold) defect in CD4 downregulation, whereas W23 was completely conserved, highlighting its critical role in both major functions of Vpu [36], [38]. Two other mutations in the transmembrane domain led to a specific loss of anti-tetherin function: I9M and I16E. Whilst not forming part of the “alanine face” of Vpu, these polar or charged residues are adjacent and may impact upon accessibility of the tetherin binding interface.

Interestingly, mutations occurring at the DSGNES β-TrCP binding site that occurred between the two phosphorylated serines, N55H (n = 2) and E56G (n = 1), were highly specific and deleterious for anti-tetherin activity, but were functional for CD4 downregulation. Mutations at or outside these phosphorylated serines, as described earlier, had severe effects on both functions and behaved essentially as the S52,56A mutant used as a functionally defective control in the function assays. Since β-TrCP is essential for CD4 downregulation by Vpu [39], these data suggest that there is a separable element to the function of this region that is independent of SCF E3 ubiquitin ligase recruitment.

As we had thoroughly examined 304 Vpus for CD4 downregulation, and tetherin counteraction, we also decided to test those defective for counteraction of both tetherin functions for their ability to downregulate cell-surface tetherin expression (**Figure S5**). We found the majority of Vpus defective for virus release maintained the ability to downregulate tetherin, possibly due to the majority of the mutations tested mapping to the DSGNES, which has residual function for tetherin downregulation [36], [40], and to the second cytoplasmic helix, previously suggested to have intermediate impact on internal tetherin sequestration [41].

### Further investigation of Vpu proteins containing lesions in the DSGNES β-TrCP binding motif

**Figure 5:**
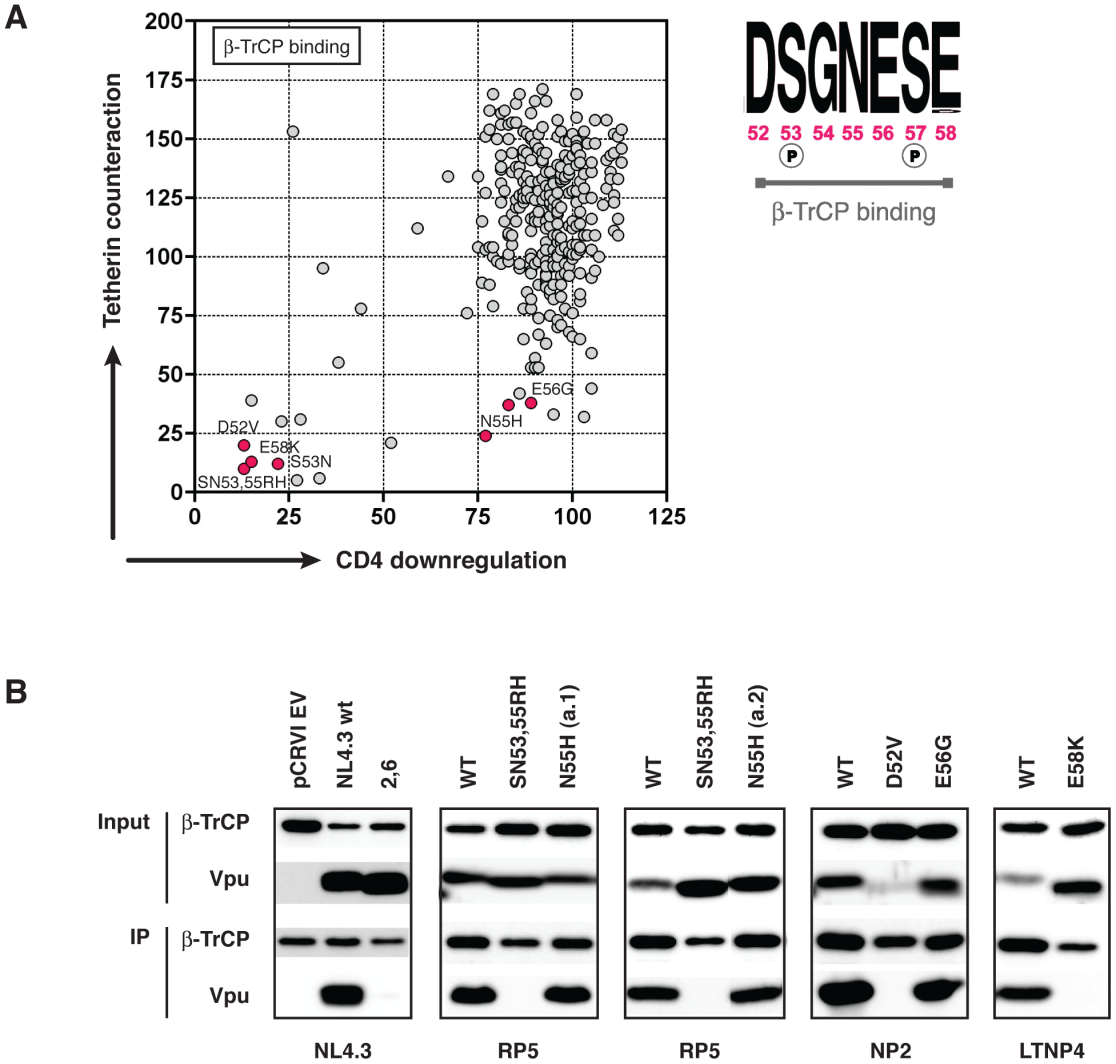
Further investigation of the β-TrCP binding properties of the N55H and E56G Vpu mutants. (**A**) We identified seven naturally defective Vpus with mutations in the DSGNES β-TrCP binding site, or in the adjacent glutamic acid that is essential for the casein kinase II-mediated phosphorylation of serines 53 and 57. Six of these mutants were tested for their ability to bind β-TrCP in co-immunoprecipitation experiments (**B**). Vpu negative (pCRVI), NL4.3 wt (positive control) and NL4.3 S52,56A mutant (negative control) are shown for comparison. In all cases, the mutant Vpu was compared to its closest functional relative (wt). Vpus are therefore described as the wt Vpu from that individual, plus the position and type of the DSGNES mutation. 293T cells were co-transfected with pCRVI-Vpu (or EV) and pCR3.1-myc β-TrCP (or GFP) and 48 hours later lysed and immunoprecipitated with anti-myc antibody.

The dichotomy of function illustrated in **Figure 4** and **Figure S4**, wherein observed mutations of the D52, S53, and E58 lead to a severe defect in both tetherin antagonism and CD4 downregulation, whilst mutations of N55 and E56 disproportionately affected tetherin counteraction, warranted further investigation. The β-TrCP binding consensus sequence is D_p_SGxx_p_SE, where both serines are phosphorylated, and in all β-TrCP substrates other than Vpu (e.g. IκBα, β-Catenin, CDC25B), the amino acid adjacent to the glycine is hydrophobic, packing into a hydrophobic patch in the binding groove of β-TrCP. In Vpu, however, this residue is a highly conserved hydrophilic asparagine, mutation of which to histidine results in a dramatic reduction in the ability of the Vpu to counteract tetherin and promote virus release. We therefore set out to determine whether this functional defect was due to a reduced or abolished ability of the Vpu to bind β-TrCP by performing Vpu and β-TrCP co-immunoprecipitions. Using the closest functional relative from the same infected individual as a positive control, we compared the binding of all Vpus that had mutations in the DSGNES region. As expected, we observed no β-TrCP binding by Vpus containing mutations of S53, D52, and E58K. However, we observed a robust binding of β-TrCP by the N55H and E56G natural Vpu mutants (**Figure 5**), suggesting an alternative tetherin-specific defect imposed by these mutations.

### Examination of the ability of Vpus to suppress tetherin-mediated NF-κB signalling

Given the demonstrated superiority of a select few patient-derived Vpus to suppress tetherin-mediated NF-κB activation (**Figure 3**), we next tested all 304 Vpus in order to obtain both a full picture of signalling suppression in natural Vpu proteins, and also to discern potential residues in Vpu specifically involved in this function that have hitherto been uncharacterised. To date, there have been no reports of regions of Vpu required to specifically suppress tetherin signalling, although a generalised suppression of NF-κB activation upon over-expression of NL4.3 Vpu has been linked to the conserved β-TrCP binding site [42].

**Figure 6:**
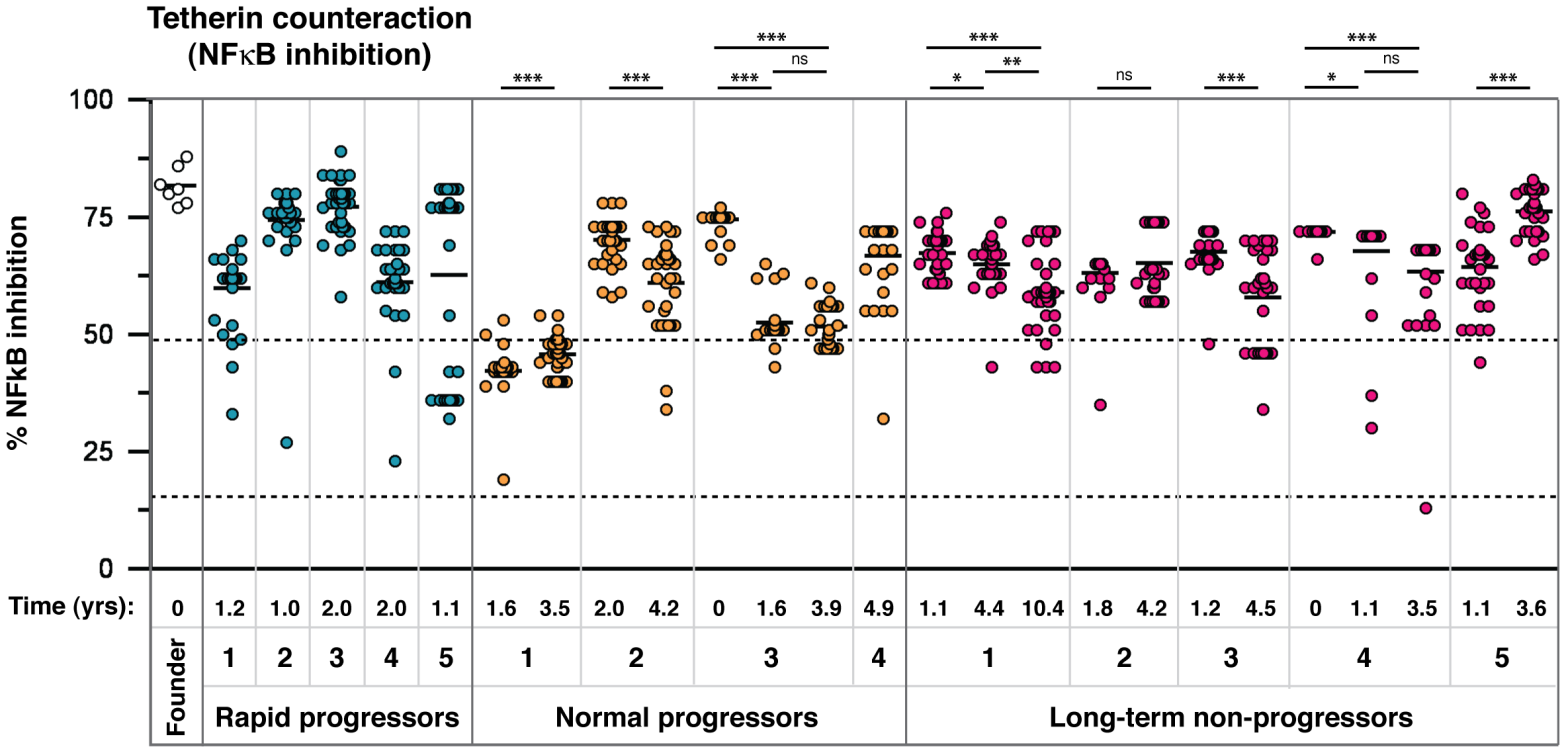
Analysis of tetherin signalling suppression by 304 natural Vpu alleles. As for Figure 2, representatives of all amino acid sequences obtained through SGA were tested in tetherin signalling suppression assays. The time point of each sample, in years post-seroconversion, is indicated beneath the graph with the patient identification and progression group. Results are shown as % reduction of maximum tetherin-mediated NF-κB activation. NL4.3, and the NL4.3 S52,56A Vpu mutant, defective for both CD4 downregulation and tetherin counteraction activity, are indicated by dashed lines (at 49 and 17%, respectively). Each symbol represents the average of a minimum of three independent experiments, weighted to represent the allele frequency of each variant, to give an overall proportional representation of function per time point. Means for overall Vpu function for each time point are shown as short horizontal lines. Significant differences between time points from each individual are indicated by asterisks. Briefly, the assays were performed as follows: 50 ng of tetherin or empty vector constructs were co-transfected with 50 ng of pCRVI-Vpu or empty vector, and 10 ng of an NF-κB-dependent firefly luciferase reporter construct and 5 ng of a control renilla luciferase construct. 24 hours later, cells were lysed and the luciferase activity determined. Results are displayed as % reduction of luciferase activation in the presence of Vpu relative to the mean maximum activity in the absence of Vpu.

As with tetherin antagonism, there was a broad range of signalling-suppressive function, with some time points containing clusters of inferior Vpu function (**Figure 6**). Interestingly, in several individuals, including those from whom a seroconversion sample was available, signalling suppression was higher in the early time point and significantly declined over time (**Figure 6**; NP2, NP3, LTNP3, LTNP4). In these individuals tetherin antagonism for virus release increased over time (**Figure 2**), indicating a trade off between the two elements of tetherin counteraction, and that the ability of Vpu to suppress tetherin-mediated signalling is not wholly determined by the physical counteraction of tetherin.

### Structure-function analyses of *vpu* alleles: Comparison of two tetherin counteractivities

**Figure 7:**
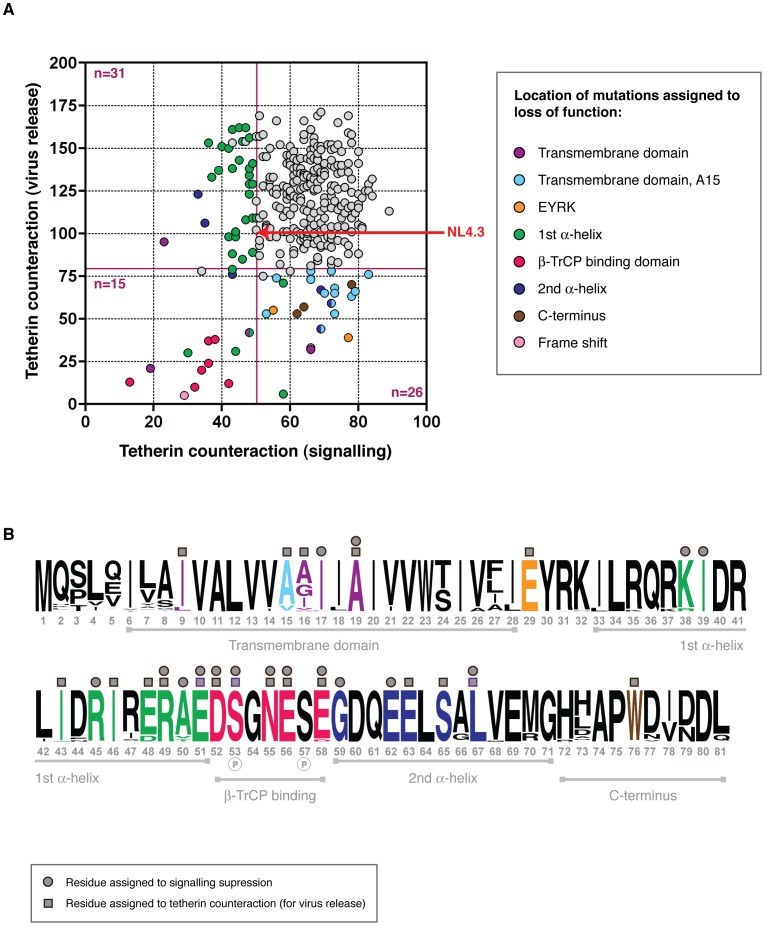
Structure-function analysis of Vpus for counteraction of two tetherin activities. (**A**) As for Figure **4**, but functional profiles of each Vpu is shown according to its ability to counteract tetherin to promote virus release, and to suppress NF-κB activation by tetherin. Tetherin counteraction (virus release) is measured relative to NL4.3 Vpu (100%), whereas suppression of signalling is presented as % reduction of NF-κB activation relative to the negative control. Defective/sub-optimal Vpus are defined as 0–81% for tetherin counteraction, and 0–50% for signalling suppression. Cutoffs are indicated by dark red solid lines. Vpus were categorised according to whether they were defective for tetherin counteraction for virus release (n = 31), signalling suppression (n = 26), or both (n = 15), then compared to their closest functional relatives and to the Vpu population as a whole, to deduce the amino acid changes responsible for the defect. Each defective/sub-optimal Vpu is coloured according to the location of the inactivating mutation, as detailed in the key, and then highlighted in (B). (**B**) As for **Figure 4B**, but in contrast, here we have only indicated residues identified in these analyses involved in tetherin counteraction for virus release (dark grey squares above logo plot), tetherin counteraction for signalling suppression (dark grey circles above logo plot), or both, rather than also indicated residues previously identified in the literature.

Since the Vpu profiles of 14 infected individuals were not similar when compared for their ability to antagonise tetherin to promote virus or to suppress signalling, we investigated whether differences in these two activities could be assigned to specific amino acid changes not critical for the promotion of virus release. Taking the same approach as that used to compare tetherin antagonism and CD4 downregulation, functional profiles of all 304 Vpus were plotted (**Figure 7**). As is evident from **Figure 6**, we observed no correlation between the ability of Vpu to physically antagonise tetherin and its ability to suppress tetherin signalling. Mutations that affected both functions were found in the DSGNES motif, a frameshift, the highly conserved R49 and E51 in the first alpha helix and A19 in the transmembrane domain. Interestingly, there were a considerable proportion of Vpus that were still able to counteract tetherin for virus release, but had defects in signalling suppression. These mapped to three conserved residues: G59 and E62 in the second alpha helix of the cytoplasmic domain, and R45 in the first alpha helix. A cluster of Vpus had defects that mapped to A50V or -T changes, which accounted for the suboptimal activity of the majority of Vpus isolated from NP1 (**Figure 6**). One more tetherin signalling-specific defect, I17T, was also defective for CD4 downregulation; all others were only defective for this particular function (see **Table S1**). One Vpu with a major defect in tetherin antagonism, II43,46SL, was still able to reduce NF-κB activation, and more modest mutants such as E29K, which were also defective for CD4 downregulation, maintained the ability to reduce tetherin signalling. Minor but common tetherin antagonism defects, A15V or –T, had no impact on the ability of these Vpus to suppress tetherin signalling.

The ability of many Vpus to suppress tetherin signalling independently of their ability to promote virus release prompted us to investigate whether Vpu possessed a global NF-κB suppression activity, mediated through the sequestration of β-TrCP, as previously reported [42], [43]. To test this we looked at the effect of increasing concentrations of various Vpu proteins on the activation of NF-κB by MAVS, a central adaptor protein in NF-κB activation pathways triggered by RIG-I-like RNA sensing receptors.

**Figure 8:**
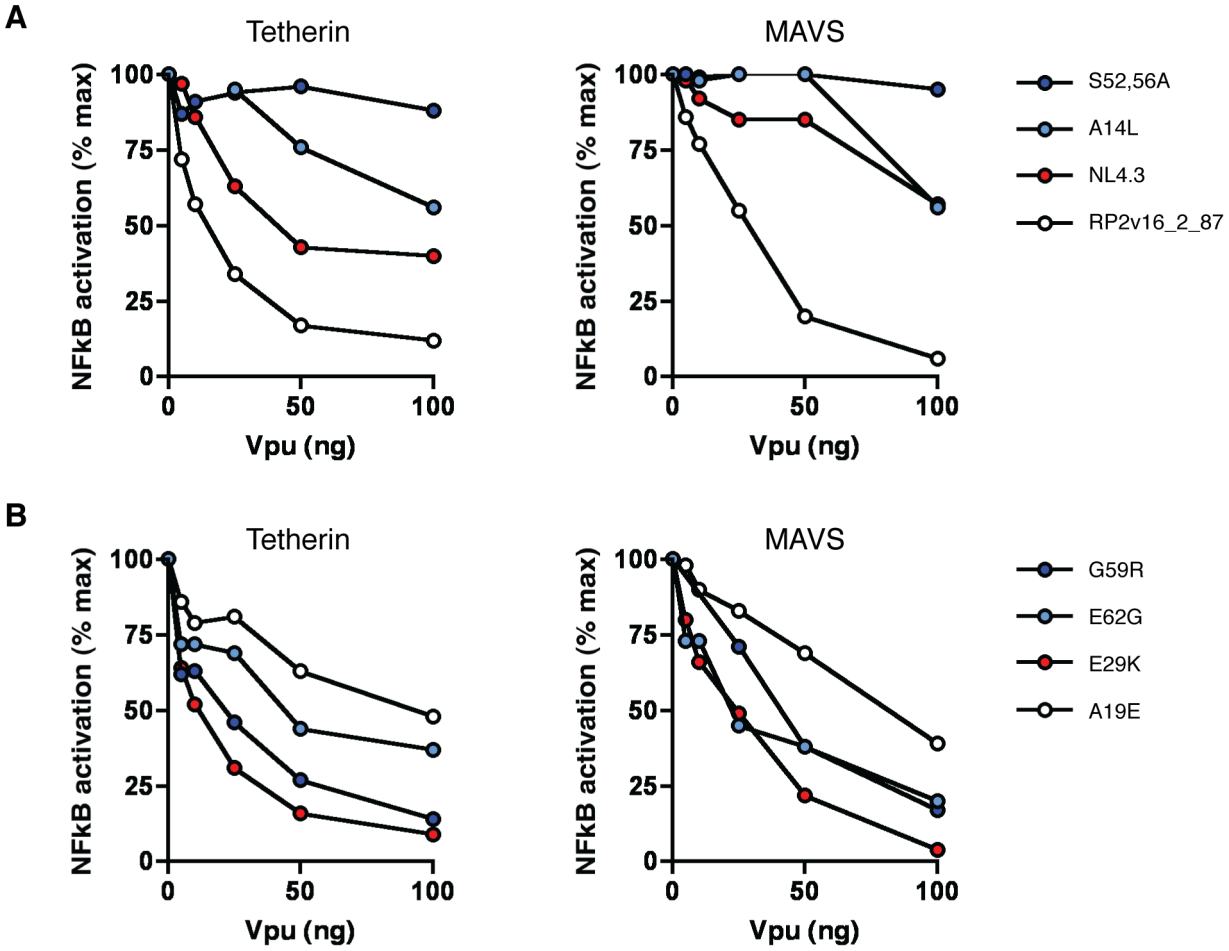
Ability of Vpu to suppress NF-κB activation. (**A**) NL4.3 Vpu, NL4.3 A14L and S52,56A mutants, and the highly active patient-derived Vpu RP2v16_2_87 were tested at a range of concentrations for their ability to counteract tetherin-mediated NF-κB activation (left panel) and MAVS-mediated NF-κB activation (right panel). Transient NF-κB activation assays were performed by transfecting 293 cells with 50 ng pCR3.1 human tetherin or 10 ng pCR3.1 MAVS alongside 0, 5, 10, 25, 50 and 100 ng of each pCRVI-Vpu. Fold activation of a luciferase NF-κB reporter gene is calculated relative to a GFP control in the presence of increasing concentrations of Vpu, and results are presented relative to the mean signal obtained in the absence of Vpu (% max). (**B**) As for (A), but with patient-derived Vpus with defects in tetherin signalling suppression but not promotion of virus release (G59R, E62G); patient-derived Vpu with defects in promotion of virus release but not suppression of signalling (E29K); and patient-derived Vpu with defects in both tetherin signalling suppression and promotion of virus release (A19), all identified in **Figure 7**.

First we compared the ability of a highly active patient-derived Vpu (RP2v16_2_87) to counteract tetherin- and MAVS-mediated NF-κB activation, along with NL4.3 and known mutants thereof (**Figure 8**). RP2v16_2_87 Vpu was highly effective in suppressing NF-κB activation by both tetherin and MAVS, with an 88% and 94% reduction in signalling by both molecules, respectively, at the highest concentration tested (**Figure 8A**). NL4.3, in contrast, showed a weaker but dose-dependent ability to suppress tetherin signalling, but was severely defective for the inhibition of MAVS signalling, with an effect seen only at the highest concentration of 100 ng. The S52,56A NL4.3 mutant, unable to interact with β-TrCP, had no signalling-suppressive activity against either tetherin or MAVS, whereas the A14L tetherin binding mutant was able to partially inhibit NF-κB activation by both proteins at higher concentrations, consistent with the notion that Vpu mediates a concentration-dependent generalised inhibition of NF-κB activation that is independent of its ability to physically counteract tetherin.

We next examined patient-derived Vpus that showed differential ability to counteract tetherin to promote virus release and to suppress signalling. Of these, an E29K mutant that was defective for both tetherin counteraction and CD4 downregulation was highly active in suppressing both tetherin and MAVS-mediated NF-κB activation. Conversely, G59R, E62G and A19E mutants were all impaired, to various degrees, for their ability to suppress both tetherin- and MAVS-mediated activation of NF-κB.

### Positive selection in Vpu sequences from natural infection

To investigate whether certain amino acid changes were selected for within a given viral pool, either due to immune escape or functional advantage, we performed positive selection analyses on the complete *vpu* sequence sets from each infected individual. Overall, the *vpu* gene was found to be under purifying selection (mean dN/dS ranging from 0.20 to 0.72 across individuals), with the identification of several individual amino acids under positive selection pressure (**Figure S6**). For the purposes of the population-level positive selection analysis, only the part of *vpu* that does not overlap with the *env* reading frame (codons 1–54) was included in the analyses. For the separate patient analyses, positions that were found to be under positive selection that fell in the overlap were assessed on an individual basis (for details see Materials and Methods). Few of the codon positions under positive selection were common to more than one individual, although positions in the N-terminus and transmembrane domain of the protein were frequently selected for (see **Table 2**, LTNP 1, 3, 5, NP 1, 2, RP 1 and 2). We found no positively selected sites associated with patterns of disease progression.

**Table 2:** Genetic distance, positive selection and predicted T cell epitopes.

Subject	Sequence number (total)	Time points	Mean genetic distance (aa)^1^	Sites under positive selection^2^	Sites under transient selective pressure^3^	Predicted T cell epitopes^4^
**LTNP 1**	105	3	**0.058**	**9**	2,4,7,9,16	4-LEILALVAL-12 B*1801
						24-SIVALEYRR-32 A*3101
						26-VALEYRRILR-35 A*3101
						32-RILRQRKIDR-41 A*3101
						41-RIINRIRER-49 A*3101
						62-EELSGLVEM-70 B*1801
						68-VEMGHHAPW-76 B*1801
**LTNP 5**	67	2	**0.043**	7,27,73		9-IVALVVAAI-17 A*3201
						17-IIAIVVWSI-25 A*3201
						21-VVWSIVLIEY-30 A*1101
						24-**SIVLIEYRK**-32 A*1101
						38-KIDRLIDRI-46 A*3201
						61-QEELSALVEM-70 B*4402
						68-VEMGHDAPW-76 B*4402
**LTNP 3**	59	2	**0.035**	**16**	16	4-LEIVSIVAL-12 B*4403
						5-EIVSIVALV-13 A*2601
						61-EELAALVEM-70 B*4403
**RP 3**	36	1	**0.033**	5		4-LAILAIVAL-12 B*3501
						22-VWSIVLIEY-30 A*2902
**RP 4**	33	1	**0.023**			NP
**NP 2**	67	2	**0.022**	4,**41**,**81**	81	5-QILAIVALV-13 A*0201
						13-VVAGIIAIV-21 A*0201
						17-IIAIVVWTI-25 A*0201
						66-ALVERGHLA-74 A*0201
**RP 2**	35	1	**0.020**	**2**	2	NP
**RP 5**	34	1	**0.019**			NP
**NP 1**	76	2	**0.016**	5		NP
**NP 3**	112	3	**0.015**			17-IIAIVVWTI-25 A*2402
						68-VEMGHHAPW-76 A*4402
**NP 4**	30	1	**0.015**			NP
**RP 1**	36	1	**0.011**			NP
**LTNP 2**	66	2	**0.008**			5-VILAIVALV-13 A*0201
						13-VVAIIIAIV-21 A*0201
						17-IIAIVVWTI-25 A*0201
						62-EELSALVEM-70 B*1801
						68-VEMGHRAPW-76 B*1801
**LTNP 4**	95	3	**0.004**			NP
**All**	851			**2,4,7,16**	2,4,7,15,16,47	

Subjects are shown in descending order of those with the highest mean genetic distance, with positive selection occurring at the population level (all sequences), shown in the bottom row of the table.

^1^ Mean substitutions/site for all time points per subject. Numbers are shown for the 1–2 year time point for each individual, since this is the one time point represented in all individuals, with the exception of NP 4, for whom the 4.9 year time point was the only one available.

^2^ Sites listed in bold type were picked up by more than one method (FUBAR plus SLAC or FEL); those in standard type were indicated by FUBAR alone.

^3^ Sites indicated by MEME.

^4^ The majority species for each time point was entered into an online T cell epitope prediction tool tailored to the HLA type of the individual. Only epitopes predicted by more than one method are shown, and in order of where they occur from N- to C-terminus of Vpu. Numbering indicates the amino acid start and end positions. Where more than one similar epitope was predicted, for example two epitopes overlapping the same region but of 9 and 10 amino acids, the one with the highest predicted binding affinity is shown. Peptides in bold type have a high predicted affinity (0–50 nM); those in regular type have medium predicted affinity (51–500 nM).

NP = none predicted.

### T cell-mediated immune pressure drives the variation seen in Vpu

Alignments of the amino acid sequences from each plasma sample show a regional clustering of mutations indicative of immune pressure, with positions undergoing positive selection often falling within these areas. We speculated that the regions of concentrated variation might coincide with T cell epitopes, previously poorly characterised specifically for Vpu, and that immune escape was principally driving the variation in the *vpu* gene. To ascertain the CD8+ T cell epitope potential of the Vpu sequences, the majority Vpu sequence from each time point was entered into a T cell epitope prediction algorithm (IEDB MHC Class I prediction method version 2009-09-01B), tailored to the Class I HLA haplotype of the corresponding infected individual (**Table S2**).

Overlaying the predicted CD8 T cell epitopes with amino acid sequence alignments demonstrates an accumulation of mutations in regions putatively targeted for presentation to CTLs, and often overlapping with sites under positive selection (**Table 2**). The co-localisation of predicted epitopes and positively selected amino acids explains the apparently random location of such residues. Furthermore, ordering individuals by genetic distance (mean nucleotide substitutions/site at time point 1–2 years) illustrates that those with the most variable Vpu repertoires also have the highest number of predicted CD8 T cell epitopes, allowing us to speculate that it is CD8 T cell pressure driving *vpu* variation, and that positive selection acting on apparently random positions is an indication of ongoing diversification within and around putative epitopes. Interestingly, in LTNP 1, 3 and 5, the individuals with the highest number of predicted T cell epitopes, a significant drop in one or both functions can be seen over time.

Of note, one of the positions undergoing positive selection in LTNP 5 was residue 73, a position previously linked to NK cell escape in KIR2DL2 individuals [12] (**Table 2**, **Tables S1** and **S2**). Upon further investigation we observed at least one change at this position, or at the associated position 70, in all but two KIR2DL2 positive individuals (LTNP1, 2, 3 and 5, NP 1 and RP 2); in contrast, these residues were invariant in all KIR2DL2 negative individuals (LTNP 4, NP 2 and 3, RP 3, 4 and 5).

**Figure 9:**
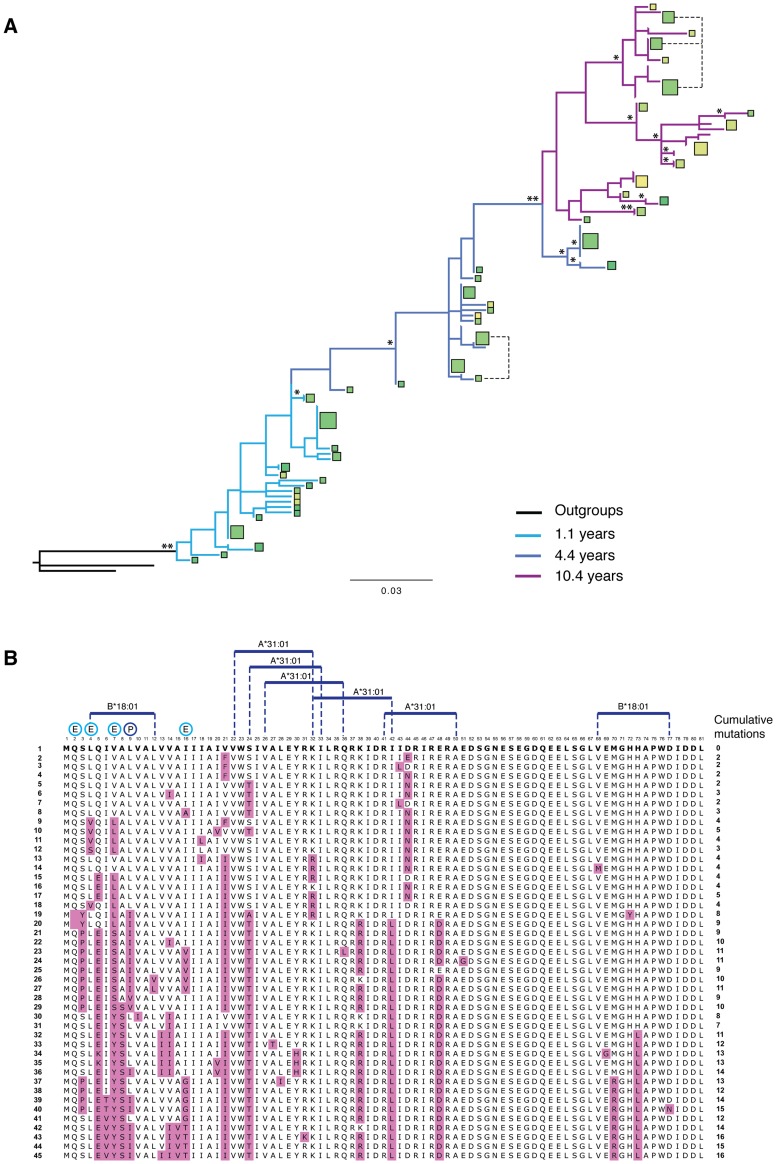
T cell epitopes and variation. Full analysis of the Vpu repertoire of one infected individual. (**A**) A phylogenetic tree of *vpu* sequences obtained from individual LTNP 1 over 3 different time points: 1.1 (light blue branches), 4.4 (dark blue branches) and 10.4 years post-seroconversion (purple branches), rooted with both NL4.3 and consensus B *vpu* sequences. Squares represent unique amino acid sequences, with the area of the square proportional to the number of Vpus with that specific sequence. The colour of the square is determined by the ability of the Vpu to counteract tetherin, based on ‘traffic light’ formatting: red being completely unable to counteract tetherin (0%), yellow having intermediate activity (75%), and green being highly active (150%). Note that in this individual most Vpu proteins had more than intermediate activity, therefore most squares are shades of green, with the darker the green the more active the protein. Branch support is indicated by one (>70%) or two (>90%) asterisks. Branch length represents the number of nucleotide substitutions per site. (**B**) An alignment of all of the unique amino acid sequences obtained from LTNP 1 and shown in (A), ordered from top to bottom according to their phylogenetic relationship. Sequence 1, to which all subsequent Vpus are compared, is that closest to the root of the tree shown in (A). The position of each sequence in the tree is indicated on the left of the alignment. Anti-tetherin activity is indicated by colour, as shown in (A). Substitutions from the ancestral sequence are highlighted in the alignment in purple, with the tally of cumulative mutations indicated on the right of the alignment. Predicted high affinity (0–50 nM) T cell epitopes, tailored to the HLA haplotype of the infected individual, are indicated above the alignment. Positions undergoing positive selection are denoted by a “P” above the alignment, with residues undergoing episodic selection (indicative of immune pressure) indicated by “E”.

To investigate further the association between predicted T cell epitopes, immune escape and Vpu variation in more detail, we selected the *vpu* repertoire with the highest genetic diversity (LTNP 1), and compared function and sequence changes over time with predicted T cell epitopes and sites undergoing transient or pervasive selection (**Figure 9**). Despite cumulative mutations occurring in 20% of the protein (16 of 80 amino acids, excluding start and stop codons), pervasive or episodic selection acting at five positions (i.e. codon positions 2, 4, 7, 9 and 16), and predicted high affinity T cell epitopes spanning the bulk of its length, every Vpu isolated from this individual was deemed functional by our classification.

## Discussion

Using single genome sequencing we have carried out a full characterisation of the sequence and function of the HIV-1 *vpu* gene throughout infection, and demonstrate that the Vpu protein has a considerable capacity for diversification and adaptation, consistent with it being one of the most variable regions of the HIV-1 genome [44]. In the face of predicted CD8 T cell pressure and significant sequence variation, it is able to maintain function regardless of disease stage or severity, with no indication of hierarchy of function.

Vpu function is strictly maintained throughout infection, as shown by fully functional Vpus obtained from transmitted/founder viruses, from seroconversion time points and from viruses isolated more than 10 years post-infection. All three functions tested – CD4 downregulation, tetherin counteraction for virus release, and inhibition of tetherin-mediated NF-κB activation – were maintained, with the vast majority of proteins (96.7%) active in at least one function. Of the ten Vpu proteins defective for all three functions, none were found in subsequent time points, suggesting that seriously defective variants do not persist over time. More minor defects in a single function did persist over time in certain individuals, for example those impaired for signalling suppression seen in NP 1, but the real impact of modest defects *in vivo* is difficult to gauge. It is also possible that, for suppression of signalling, strict maintenance of function is more important at early stages of infection, and declines with time, as seen most notably in NP 3.

For tetherin counteraction, maintenance of function reflects other reports of immunodeficiency viruses responding to the pressure exerted by tetherin, including the recent characterisation of a HIV-1 group N Vpu that has evolved to become an efficient tetherin antagonist [13], the demonstration of acquisition of tetherin antagonism in the Env proteins of *nef*-deleted simian immunodeficiency viruses [45], and the reacquisition of tetherin counteractivity in Nef following experimental infection of chimpanzees with HIV-1 [46]. Furthermore, studies of HIV-1/hepatitis C-co-infected individuals have demonstrated that, following treatment with pegylated interferon, an increased expression of tetherin in peripheral CD4+ T cells correlates with a significant reduction of HIV-1 viral load, with some indication of compensatory mutations in Vpu [31]. Selective pressure exerted by tetherin is indicative of its multiple antiviral effects: not only its ability to physically prevent the release and spread of virus particles, its role as a pattern-recognition receptor and potential enhancer of antigen presentation [47], but also the potential for enhanced antibody/complement opsonisation and NK cell recognition that may be downstream consequences of virion restriction. This is manifest in the observation that populations of sub-optimal Vpus with specific defects in tetherin counteraction in early time points, such as the group of Vpus with A15V in NP2 and LTNP 3, are found in significantly lower frequencies at the subsequent time point, indicative of selection against Vpus with inferior tetherin binding activity.

Our previous work demonstrates the ability of tetherin to induce an NF-κB-mediated proinflammatory signal [7], and here we thoroughly examine the ability of 304 primary Vpus to counteract tetherin signalling. The suppression of tetherin-mediated NF-κB activation was observed at a high level across the patient groups particularly at early time points, with the notable exception of NP1. However, while the majority of Vpus were superior to NL4.3 Vpu in both functions, there was no direct relationship between the ability to promote virus release and the ability to suppress signalling; in some individuals an increase in the former function over time was mirrored by a decrease in the latter. This prompted us to investigate whether signalling suppression, particularly by those Vpus with defects in direct tetherin antagonism, was in part due to a previously recognised [42], [43], and more recently expanded [48], intrinsic ability of Vpu to globally suppress NF-κB activation. This ability of Vpu is primarily driven by its binding of β-TrCP, a component of the SCF E3 ubiquitin ligase complex that is required for the ubiquitination and degradation of IκB, and subverted by Vpu for the degradation of its target proteins [49]. Indeed, we confirmed that NL4.3 Vpu was able to suppress NF-κB activation by both tetherin and MAVS, but only when overexpressed, i.e. at levels unlikely to be found in an infected cell. Our patient-derived Vpus, however, were able to reduce NF-κB activation even at lower levels of expression, with a complete ablation of signalling occurring at higher Vpu concentrations, suggesting that this may indeed be an important role of Vpu *in vivo.* Furthermore, the observation of a significant decline in signal-suppressive function over time in several individuals, in contrast to the other two functions examined, as well as the high activity observed in founder virus-derived Vpus, may be indiciative of this activity being most important in early stages of infection.

In addition to fully characterising natural *vpu* alleles, the secondary aim of this study was to identify determinants of the protein that are required for one or all functions. To the previously precisely defined regions involved in tetherin antagonism (A15, A19, W23; E63, L67, V68 [4], [36], [37]), we contribute I9, A16, E29, II43,46, E48, R49, E51, N55, E56 and W76. At positions recently highlighted by McNatt and colleagues to interact with tetherin (I5, A8, V21, V22, V26, I27, I28), we see variation amongst our patient-derived Vpus and encountered no changes here that impacted on tetherin counteractivity. However, an I9M mutation that was attributed to a serious defect in tetherin antagonism (**Figure 4**), is adjacent to a residue proposed to interact with tetherin [41]. V21 and V22 residues, also indicated as interacting residues [41], we found had more influence on CD4 downregulation and little impact on tetherin counteractivity.

The N55H and E56G mutations are particularly interesting, since they occur within the DSGNES motif containing the phosphorylated serines that mediate interaction with β-TrCP, and yet these particular changes leave CD4 downregulation largely intact. While previously suggested to impact on tetherin antagonism [50], the lesser impact on CD4 downregulation promted us to investigate this further. We were able to show that Vpus with N55H or E56G mutations are still able to bind β-TrCP. Since other mutations in the DSGxxS diserine motif (e.g. D52V, S53N, E58K) had severe effects on β-TrCP binding and CD4 downregulation, this suggests a dual function of this region in accordance with previous demonstrations that β-TrCP is not strictly required for tetherin trafficking by Vpu [51]. It is possible that this reflects an as yet unidentified Vpu co-factor, or involves facilitating access to either of the two cytoplasmic alpha helices. In this respect, it is interesting to note that acidic-dileucine motifs, such as the ExxxLV motif of Vpu, have previously been associated with phosphorylation in the trafficking of the cation-independent mannose-6-phosphate receptor [52].

Examination of the ability of 304 different Vpu proteins to suppress tetherin-mediated NF-κB activation revealed a number of previously uncharacterised residues important for this function. Residues that were important for inhibition of tetherin signalling, but not for the other two tested functions, mapped to G59 and E62 in the second alpha helix, and A50 in the first alpha helix. Vpus containing G59 and E62 were likewise partially defective in inhibiting NF-κB activation by MAVS when tested over a range of concentrations, therefore indicating that this little characterised activity of Vpu involves residues in Vpu beyond that of the DSGNES β-TrCP binding site. Conversely, Vpu proteins that were defective for tetherin counteraction (e.g. E29K), maintained the ability to suppress tetherin signalling, and NF-κB activation in general, presumably through possessing an intact β-TrCP binding site. The fact that this ability of Vpu involves regions of the protein beyond the β-TrCP binding site, may indicate that the mechanism of signal suppression is more complex than the sequestration of β-TrCP.

To the more elusive residues involved in CD4 downregulation, including V21, S/T24 [38] and L67 [53], we add I17, V22 and I39, in addition to the E29, I43, I46 and R49 residues also found to affect tetherin counteraction. We found no mutations in the second alpha helix that may be attributed to CD4 interaction, as previously suggested [54], although this may be due to more conservation in this area and therefore a lack of mutants with potential functional defects. V22 (V21 in NL4.3 Vpu) has previously been reported to have a mild effect on CD4 downregulation, as we show here [38]. I17 and I39 are highly conserved residues, and to our knowledge have not previously been implicated in CD4 downregulation, although they do fall within the transmembrane domain and first alpha-helix, two regions other than the DSGNES β-TrCP binding region previously reported to be important for CD4 downregulation [4], [55].

It is perhaps surprising, considering that few other members of the immunodeficiency viruses have this capability, that the CD4 degradation activity of virtually all Vpus tested is so strictly maintained. The reasons for this are unclear; all known immunodeficiency viruses possess an activity in Nef that induces the endocytosis of CD4 from the infected cell surface, with only the HIV-1 groups M, O and P, and the SIVcpz viral lineage employing a further Vpu-induced CD4 degradation step in the ER. Yet, there is little doubt from our data that the degradation of CD4 in the ER is absolutely required by HIV-1 *in vivo*, and there is no suggestion that any redundancy of function exists between Vpu and Nef, or that a reduction in this function is tolerated over time. HIV-1 envelope affinity for CD4 is reportedly higher than that of tested SIV envelope proteins, thus it has been proposed that Vpu is required to effectively chaperone the Env protein through the ER, thus avoiding this high-affinity interaction and subsequent loss of Env integrity [56].

The importance of other recently reported functions of Vpu remains to be explored. Vpu plays a further role in the modulation of immune recognition of the infected cell through downregulating the NK cell activating ligand NTB-A [9], and through reducing the surface expression of PVR and CD1-d [10], [11]. Thus far, studies comparing the effects of Vpu *in vitro* and in humanised mice have demonstrated a clear effect of Vpu on CD4 and tetherin, with modest effects on NTB-A and CD1d [13], [57], [58]. The mechanisms also appear to differ, with the serines central to CD4 downregulation and tetherin counteraction not required for downregulation of cell surface NTB-A [9]. It will be interesting to ascertain whether the minority of non-functional alleles isolated in this study have residual activity against either of the recently characterised targets, and whether they are present in circulating virus strains because they modulate the recognition of the infected cell by NK or NK-T cells. We also see evidence of Vpu's immunomodulatory function in signature residues at its C terminus (amino acids 70 and 73) previously linked to NK cell escape. Indeed, in KIR2DL2 positive individuals we detect ongoing variation at these positions; interestingly, this was most apparent in long-term non-progressors, and in one such individual we observe positive selection acting at position 73, in accordance with the amino acid position associated with NK cell escape characterised by Alter et al [12].

Predicted CD8 T cell pressure coincides with positions of the protein we detect as being under positive selection. In at least one individual (LTNP 5) we see evidence of escape from a high affinity T cell epitope at the later time point. In others, mutations occur in flanking residues of the peptide, potentially affecting peptide processing. In the two individuals with the highest variability and highest predicted T cell pressure (LTNP 1 and 5), we see a significant reduction of overall tetherin antagonism over time; however, as discussed above, the levels do not drop below that of NL4.3 and thus we predict would attain the threshold of activity required to manage tetherin *in vivo*.

The demonstration of continuous pressure on the virus to maintain high levels of Vpu function, and our detailed analysis of Vpu sequence-function relationship, puts forth strong support for the development of antiretroviral compounds targeting Vpu, whilst providing an excellent resource for the future study of disease-relevant Vpu alleles. In particular, we provide data on the regions of Vpu common to two or more functions, and those that appear to be specific to one. We demonstrate that gross defects are not tolerated, making Vpu a potential target for drug development; yet, we stress the importance of assessing multiple parameters of accessory gene function. In this respect, replication in culture assays may be misleading as to the potency of a compound, and may call for validation in new animal model systems [59]. Furthermore, this study highlights the importance of using representative primary HIV-1 proteins for the purpose of vaccinology and drug discovery. Passage in culture, in the absence of pressure to maintain certain accessory gene functions, can lead to a lack of potency in several of these proteins, including Vpu. Thus, there are clear pitfalls that come with using potentially unrepresentative, albeit historically well characterised, proteins derived from laboratory-adapted viral strains such as NL4.3.

## Materials and Methods

### Preparation of RNA and cDNA from clinical samples

Plasma samples were obtained from 14 treatment-naïve individuals (i.e individuals that had not received treatment either during or prior to sampling) enrolled at the Chicago Clinical Research Site for the Multicentre AIDS Cohort Study (MACS). HIV-1 disease progression was defined by time to AIDS and included 5 long-term non-progressors (defined as >10 years from seroconversion to onset of AIDS), 4 normal progressors (5–9 years to onset of AIDS), and 5 rapid progressors (<5 years to onset of AIDS) [32]–[34]. Where possible, a 1–2 year and 3–4 year post-infection time point was obtained per individual. Progression status was assigned retrospectively, as standard in the MACS. For the RPs in this study, early disease progression precluded the availability of a 3–4 year time point. For NP 3 and LTNP 4, additional seroconversion time point (0 years) was also analysed, and for LTNP 1 a 10.4-year time point.

The volume of plasma equivalent to 10,000 copies of viral RNA (based on standard clinical viral load measurements) was first centrifuged to remove cellular debris (5,400*×g* at 4°C for 10 mins), then the virions were pelleted (25,000×g at 4°C for 1 hour). Viruses derived from blood samples collected in heparin were then heparinase treated to avoid inhibition of downstream enzymatic processes. RNA isolated from the virion pellets was then transferred to standard reverse transcription reactions (Invitrogen Superscript III), using Vpu-specific outer reverse primer (see SGA section below), according to manufacturer's instructions.

### Single genome amplification (SGA)

Single genome amplification techniques were based on methods described in Palmer et al. [60] Nested PCR primers were designed to conserved regions in the *tat/rev* first exon and the *env* gene according to sequences derived form the Los Alamos National Laboratory HIV Sequence Database (forward EK5846-5869 5′-CCT AGA CTA GAG CCC TGG AAG CAT-3′; reverse EK6473-6453 5′-TTC TTG TGG GTT GGG GTC TGT-3′), with the inner primers containing standard sequencing primer sequences T7 and M13 (forward EK5972-5990 5′-TAA TAC GAC TCA CTA TAG GCA GGA AGA AGC GGA GAC A-3′; reverse EK6848-6330 5′-CAG GAA ACA GCT ATG ACC CCA TAA TAG ACT GTG AC-3′), numbered according to the HXB2 molecular clone. Viral cDNA was serially three-fold diluted from 1∶5 to 1∶405 and used as a template for multiple PCRs. We first performed 12–24 reactions at the highest dilution, and the number of positive reactions was used to calculate the cDNA dilution at which approximately 30% would be positive as predicted by the Poisson distribution. We then performed 92 PCRs at this modified dilution, and reactions yielding a product were directly sequenced with T7 and M13 primers (MWG Eurofins, Germany). Chromatograms were carefully examined for the presence of double or multiple peaks.

### Phylogenetic analyses

For each patient, complete *vpu* gene sequences were manually aligned with the software Se-Al version 2.0a11 [61]. Maximum likelihood phylogenies were reconstructed under the General Time reversible (GTR) model of nucleotide substitution, with gamma-distributed rate heterogeneity, using RaxMLGUI version 1.2 [62]. Robustness of the tree topologies was assessed by non-parametric bootstrap testing, with 1000 replicates, also performed with RaxmlGUI. Trees were edited using the software FigTree version 1.3.1 [63]. A maximum likelihood phylogeny containing sequences from all patients was also reconstructed following the same procedure.

Intra-host pairwise genetic distances were calculated using the phylogenetic package HyPhy version 2.1.2 [64]. For each alignment, nucleotide and amino acid substitution matrices were estimated under the GTR and Whelan & Goldman models respectively.

Codon-specific selection analyses were conducted via the HyPhy webserver DataMonkey [65]. Three different methods were used to identify *vpu* sites evolving under constant adaptive pressure: Single Likelihood Ancestor Counting (SLAC), Fixed Effects Likelihood (FEL) and Fast Unbiased Bayesian Approximation (FUBAR). For each patient, estimations were conducted under the best fitting model of nucleotide substitution, as selected by the model selection procedure implemented in DataMonkey. Sites showing evidence for positive selection by more than one method at the p<0.05 (SLAC and FEL) or posterior probability >0.95 (FUBAR) significance level were included in the study. In addition, the Mixed Effects Model of Evolution (MEME) method was used to identify sites subjected to episodic selective pressures (posterior probability >0.95). Sequences were screened for recombination prior to analyses, using the Single Breakpoint Recombination (SBR) and Genetic Algorithms for Recombination Detection (GARD) methods implemented in DataMonkey. No recombination breakpoint was found at the p<0.05 significance level. Codons found to be under positive selection that were located in the region of *vpu* that overlaps with the *env* open reading frame (codons 55–81), were assessed on an individual basis as follows: a non-synonymous change in *vpu* that was synonymous in *env* was scored as positive; a non-synonymous change in both genes was impossible to reliably determine which gene the selection was acting on, therefore these cases were excluded from the results.

To identify codon-specific selective pressure on the *vpu* gene at the population level, the above-mentioned procedure was repeated on an alignment containing the unique nucleotide sequences from all patients (n = 443). For population-level selective pressure, data are presented pertaining only to the region of the *vpu* gene that does not overlap with the *env* open reading frame (codons 1–55).

### Vpu cloning and expression

Vpu repertoires from each time point were stripped of duplicates, and all unique amino acid sequences from each sample were re-amplified using the inner forward and revers primers described above modified with EcoR1 and Not1 restriction sites respectively. Products from these reactions were then cloned into an Rev-dependent HIV-1-based expression vector, pCRVI [20], to obviate the need to codon optimise the Vpu sequence [66]. The resultant plasmids were then re-sequenced, to ensure that no mutations were introduced into the *vpu* genes during the cloning process.

### Tetherin counteraction (virus release) assay

HEK293T cells were seeded at 1.5×10^5^ per well of a 24-well plate the day before transfection. Cells were co-transfected with 500 ng NL4.3delVpu provirus plasmid, or NL4.3 wild-type plasmid as a control, plus 50 ng pCR3.1-human tetherin plasmid, or pCR3.1 empty vector as a control, plus a standard input of 25 ng of pCRVI-Vpu, or pCRVI empty vector as a control. In the case of titration experiments, 5, 10, 25, 50 and 100 ng of pCRVI-Vpu were used in each assay, with the total plasmid quantity kept constant by the addition of pCRVI empty vector plasmid to a total quantity of 100 ng. Cell culture medium was removed 14 hours after transfection, and replaced with 600 µl per well. 48 hours after transfection, viral supernatants and cell lysates were harvested, and infectious virus released determined by standard HeLa-TZMbl assay and virus particle release determined by Western blot. Each Vpu was tested in a minimum of three independent experiments, and results were compared between experiments by setting the level of infectious virus released in the presence of NL4.3delVpu virus plus pCRVI-NL4.3 Vpu as 100%, and expressing the activity of the patient-derived Vpus as a percentage thereof. NL4.3 Vpu constructs with defects specifically in tetherin antagonism (A14L), or tetherin antagonism plus CD4 downregulation (S52,56A and A14L/W22A) were included in all assays as negative controls.

### CD4 and tetherin downregulation assay

HeLa-TZMbl cells were seeded at 8×10^4^ cells per well of a 24-well plate the day before transfection. Cells were co-transfected with 150 ng pCR3.1-GFP or empty vector control, and 100 ng pCRVI-Vpu or empty vector control. 24 hours after infection, cells were harvested and stained for cell surface molecule expression using a mouse anti-human CD4 monoclonal antibody directly conjugated to allophycocyanin (APC; clone RPA-T4; BD Biosciences), or a mouse anti-human tetherin monoclonal antibody (clone 3H4, Novus Biologicals) followed by an IgG2a specific anti-mouse-Alexa Fluor 633 secondary antibody (Life Technologies). Cells were then analysed for CD4 or tetherin and GFP levels using a FACSCalibur flow cytometer (Becton Dickinson) and FlowJo software (TreeStar Inc, Oregon, USA). Cells expressing high levels of GFP were gated and CD4/tetherin levels were determined as the median fluorescent intensities. Absolute downregulation levels were calculated as the percentage reduction of CD4/tetherin cell surface expression (median fluorescent intensity) in the presence of Vpu compared to in the absence of Vpu (empty vector transfection). For the purposes of comparison with the tetherin counteraction assay, the absolute level of CD4/tetherin downregulation obtained in the presence of NL4.3 Vpu was normalised to 100%, and therefore the CD4/tetherin downregulation by all other Vpus expressed as a percentage thereof. (Note that the absolute CD4 downregulation in the presence of NL4.3 Vpu was 80+/−6%).

### Signalling suppression assay

HEK293 cells were seeded at 1.2×10^5^ per well of a 24-well plate the day before transfection. Cells were co-transfected with 10 ng 3×κB-pConA-FLuc 50 and 5 ng pCMV-RLuc reporter constructs, plus pCR3.1-human tetherin plasmid, or 3.1-MAVS/IPS1/Cardif, or GFP plasmid as a control, and 50 ng of pCRVI-Vpu or pCRVI empty vector as a control. 24 hours after transfection cells are harvested and luciferase activity measured with the Dual Luciferase Reporter Assay System (Promega). Luciferase signals were normalised, and fold NF-κB activation calculated in the absence of Vpu expression. In the case of titration experiments, 5, 10, 20, 50 and 100 ng of pCRVI-Vpu were used in each assay, with the total plasmid quantity kept constant by the addition of pCRVI empty vector plasmid to a total quantity of 100 ng.

### Expression of defective Vpu proteins

All Vpus classified as defective or suboptimal for both CD4 downregulation and tetherin counteraction (i.e. 0–75% that of NL4.3 Vpu activity) were tested for expression in 293T cells by Western blot analysis using a polyclonal rabbit anti-Vpu antibody [67] kindly provided by Klaus Strebel through the NIH AIDS Reagent Program. Since this antibody is specific for the C-terminal region of NL4.3 Vpu, and the patient-derived Vpus differ considerably in amino acid sequence from NL4.3 Vpu, expression of each defective/suboptimal Vpu was compared to that of its nearest functional relative from the same infected individual. Defective/suboptimal Vpus that showed low or no expression were re-transformed, re-purified and then re-sequenced to ensure the quality of the plasmid preparation, and in all cases the expression levels before and after this process were comparable. Defects were therefore deemed to be due to natural expression defects or instability of the expressed protein.

### Immunoprecipitations

293T cells were co-transfected with 600 ng of pCR3.1-β-TrCP2 myc or GFP and 600 ng of pCRVI-Vpu or EV. 48 hours after transfection, cross-linking immunoprecipitations were performed [68]. Briefly, cross-linking was preformed on harvested cells using 0.05% HCHO, then lysed in 150 mM NaCl, 10 mM Hepes (pH), 6 mM MgCl2, 2 mM DTT, 10% glycerol, 0.5% NP40, 200 µM sodium orthvanadate and protease inhibitor cocktail. Cleared lysates were immunoprecipitated with mouse anti-myc monoclonal antibody (clone 9E10, Covance) and protein G agarose beads (Invitrogen). Cross-linking was reversed with 10 mM EDTA, 5 mM DTT and 1% SDS, and lysates and immunoprecipates were analysed by Western blot using mouse anti-myc and rabbit anti-Vpu antibodies.

### Statistics

Unpaired two-tailed T tests were used to determine significant differences between samples for the CD4 downregulation (**Figure 2A**), tetherin counteraction (**Figure 2B**) and suppression of tetherin activation of NF-κB (**Figure 6**). A two-tailed Fisher's exact test was used to determine whether Vpus containing a threonine or valine at position 15 instead of alanine decreased over time in certain individuals. Levels of significance were determined as follows: *** p<0.001, ** p<0.01, *p<0.05, ns p>0.05.

### Ethics statement

Anonymized, pre-collected plasma samples and associated clinical data used in this study were obtained from the Chicago MACS Center with the permission of the Multicenter AIDS Cohort Study/Women's Interagency HIV Study. Permission to use anonymized human plasma samples was also granted by the King's College London Infectious Disease BioBank Local Research Ethics Committee (SN-1/6/7/9). Data in this manuscript were collected by the Multicenter AIDS Cohort Study (MACS) with centers (Principal Investigators) at The Johns Hopkins Bloomberg School of Public Health (Joseph B. Margolick), Northwestern University, and Cook County Bureau of Health Services (Steven Wolinsky), University of California, Los Angeles (Roger Detels), and University of Pittsburgh (Charles Rinaldo). The Data Center is located at the Johns Hopkins Bloomberg School of Public Health (Lisa P. Jacobson). The MACS is funded by the National Institute of Allergy and Infectious Diseases, with additional supplemental funding from the National Cancer Institute. UO1-AI-35042, UM1-AI-35043, UO1-AI-35039, UO1-AI-35040, UO1-AI-35041. Website located at http://www.statepi.jhsph.edu/macs/macs.html.

## Supporting Information

Figure S1 **Phylogenetic trees of** ***vpu*** **sequences obtained from 14 HIV-1-infected individuals.** Maximum likelihood phylogenies of *vpu* nucleotide sequences derived from 14 HIV-1-infected individuals. Bootstrap supports (% confidence) are shown at the base of the branch for each individual. Branch lengths indicate the number of nucleotide substitutions per site. The trees are rooted against NL4.3 and consensus B *vpu* sequences (black branches). Where multiple time points were available from one individual, earliest samples are depicted in turquoise circles; dark blue squares for intermediate time points; and purple stars for the later time point. (**A**) LTNP 1 (1.1, 4.4 and 10.4 yrs); (**B**) LTNP 2 (1.8 and 4.2 yrs); (**C**) LTNP 3 (1.2 and 4.5 yrs); (**D**) LTNP 4 (0, 1.1 and 3.5 yrs); (**E**) LTNP 5 (1.1 and 3.6 yrs); (**F**) NP 1 (1.6 and 3.5 yrs); (**G**) NP 2 (2 and 4.2 yrs); (**H**) NP 3 (0, 1.6 and 3.9 yrs); (**I**) NP 4 (4.9 yrs); (**J**) RP 1 (1.2 yrs); (**K**) RP 2 (1.0 yrs); (**L**) RP 3 (2.0 yrs); (**M**) RP 4 (2.0 yrs); and (**N**) RP 5 (1.1 yrs). The phylogenies of LTNP 3 and NP 1 were reconstructed after exclusion from the alignment of polymorphic residues showing evidence of reversion to wild type over time (codon positions 37 and 22 for LTNP 3 and NP 1 respectively), causing the artefactual clustering of late sequences closer to the outgroups than to those from the earlier time points.
(PDF)

Figure S2 **Genetic distance vs sequence number.** Mean and maximum genetic distance values (GD; nucleotide substitutions per site) for each clinical sample used in this study were plotted against the total number of *vpu* sequences obtained per sample, as shown in **Table 1**. R^2^ values are shown next to the slopes. A lack of correlation between the parameters is consistent with the *vpu* sampling number being sufficient to represent the viral population in peripheral blood at a given time point.
(TIF)

Figure S3 **CD4 downregulation and tetherin counteraction of only multiple variants.** Data from **Figure 2** for CD4 downregulation (**A**) and tetherin counteraction (**B**) were re-plotted after the removal of all Vpus derived from a single genome to assess the impact of potentially transient variants on the outcome of the analyses. See legend from **Figure 2** for details. Differences in function between time points were re-analysed for significance; in some cases, removal of single variants lead to all values for one time point being identical, and in these cases statistical analyses could not be applied (n/a; LTNP 3, LTNP 4).
(TIF)

Figure S4 **Detailed annotation of defective Vpu variants.** (A) Defective and suboptimal Vpus categorised according to the location in which the mutations responsible for their defects occur, with the specific amino acid change indicated for each allele. (B) Vpus with defects in both functions were checked for expression by Western blot analysis. The only construct from which no Vpu expression could be detected is marked with an asterisk, but in this case closely-related fully functional Vpus could not be detected either, suggesting a lack of antibody-reactivity.
(TIF)

Figure S5 **Ability of 30 patient-derived Vpus to downregulate cell surface tetherin levels.** NL4.3 Vpu and three mutants thereof (S52,56A; A14L; and AW14,22LA), 28 patient-derived Vpus with various severe to minor defects in tetherin antagonism (see **Figure 4** and **Figure S4**), a fully functional patient-derived Vpu (pos), and a founder virus-derived Vpu (WITO), were tested for their ability to downregulate tetherin cell-surface expression. TZMbl cells were transfected with 300 ng pCRVI-Vpu or EV and 300 ng pCR3.1-eGFP. 48 hours later cell surface tetherin levels were determined by flow cytometry. Fold tetherin downregulation was determined by comparing median fluorescent intensities of tetherin in the presence and absence of Vpu. Top panel: examples of FACS plots for EV, NL4.3 wt, S52,56A, A14L and AW14,22LA, and positive control Vpu LTNP1v4_1_67. Bottom panel: fold reduction of tetherin cell surface expression. Error bars represent standard deviation from the mean of 3 independent experiments.
(TIF)

Figure S6 **Positive/purifying selection acting on the** ***vpu*** **gene at the population level.** All 851 sequences obtained were stripped of duplicates and analysed for codon-specific selective pressure at the population level. Three independent methods were used (SLAC, FEL and FUBAR) and FUBAR estimates of dN-dS are represented for each of the 81 codons across the *vpu* gene. Sites undergoing significant positive and negative selection are highlighted in red and blue respectively (posterior probablility >0.95).
(TIF)

Table S1 **Complete sequence-function data of 304 natural Vpu proteins.** The amino acid sequences, CD4 downregulation activity and tetherin counter-activity of 304 *vpu* alleles isolated from 14 HIV-1-infected individuals are shown in Excel format. Filters have been applied to each column so that the sequences can be ordered or selected by function, individual, progression group, or by particular residues at each position. Vpus are named according to (i) the individual from whom they were derived (e.g. RP 1), (ii) the visit to the clinic when the sample was obtained, and (iii) the sequence ID. The frequency of occurrence of each particular allele in a given time point is indicated by the column headed “N = ”. Function is expressed as a percentage of that of NL4.3 Vpu, with traffic light formatting: red indicating no activity, yellow indicating intermediate, and green indicating highly active, with a continuous spectra of colour across these definitions. NL4.3 and Consensus B Vpu sequences are given at the top of the table for comparison, along with amino acid position and a schematic of the different regions of the protein (TM = transmembrane domain, H1 and H2 = first and second alpha helices, respectively. Amino acids in the patient-derived sequences that differ from that of Consensus B Vpu are highlighted in blue. Amino acids that have been assigned to loss-of-function of a given Vpu (as detailed in the text) are highlighted in red.
(XLSX)

Table S2 **KIR and HLA haplotypes of the 14 study subjects.**
(XLSX)
