# Correction: Preservation of Tetherin and CD4 Counter-Activities in Circulating Vpu Alleles despite Extensive Sequence Variation within HIV-1 Infected Individuals

**DOI:** 10.1371/journal.ppat.1004118

**Published:** 2014-04-14

**Authors:** The PLOS Pathogens Staff

The following funding source was omitted from the published article: The Multicenter AIDS Cohort Study is funded by the National Institutes of Health grant NIH 2 U01 AI035039-22.
