# Supplementary figures and images for: Preservation of Tetherin and CD4 Counter-Activities in Circulating Vpu Alleles despite Extensive Sequence Variation within HIV-1 Infected Individuals
Source: PLoS Pathog. 2014 Jan 23;10(1):e1003895. doi: 10.1371/journal.ppat.1003895 (PMC3900648; doi:10.1371/journal.ppat.1003895)

A  
LTNP 1

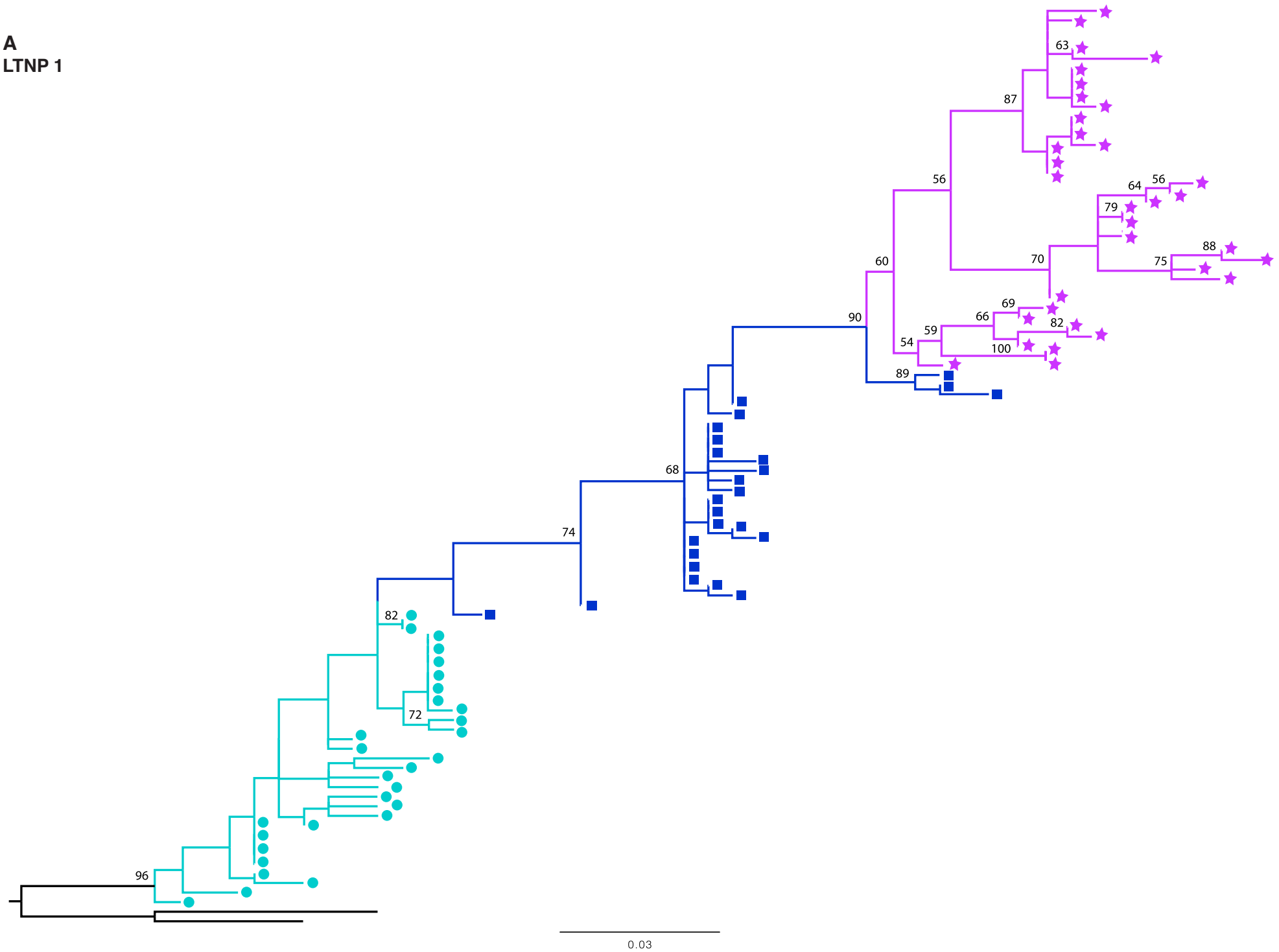

**B**  
**LTNP 2**

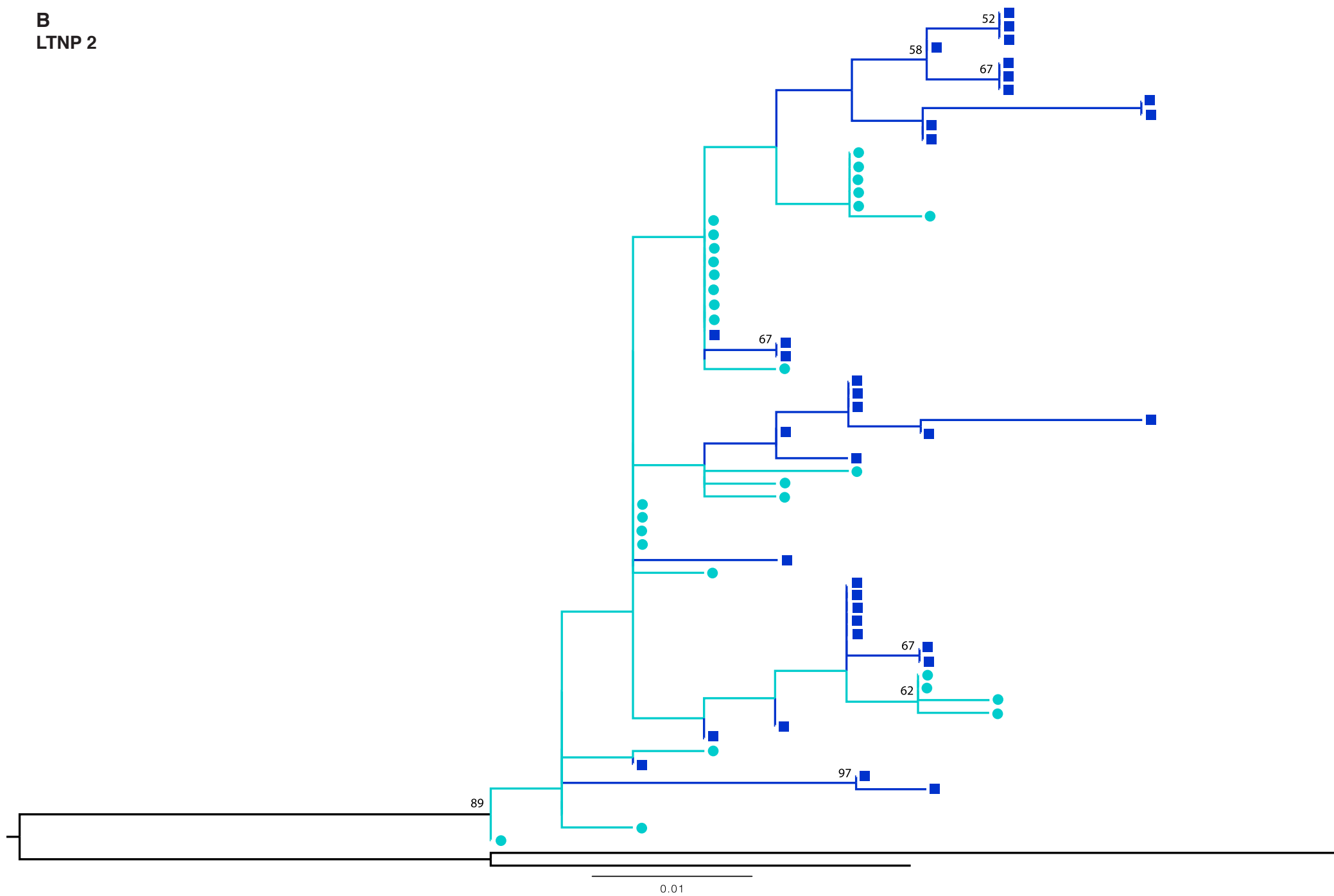

**C**  
**LTNP 3**

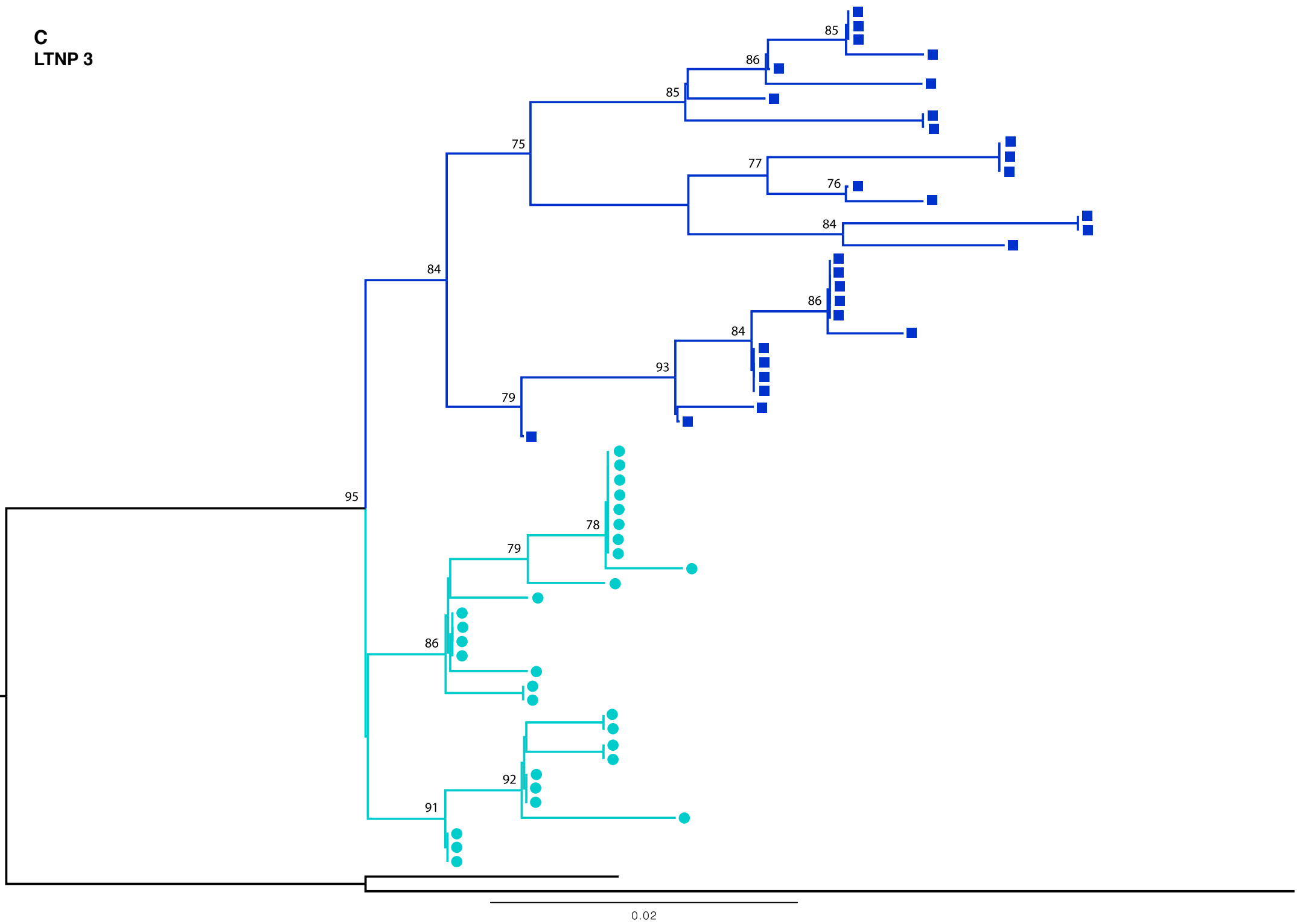

D  
LTNP 4

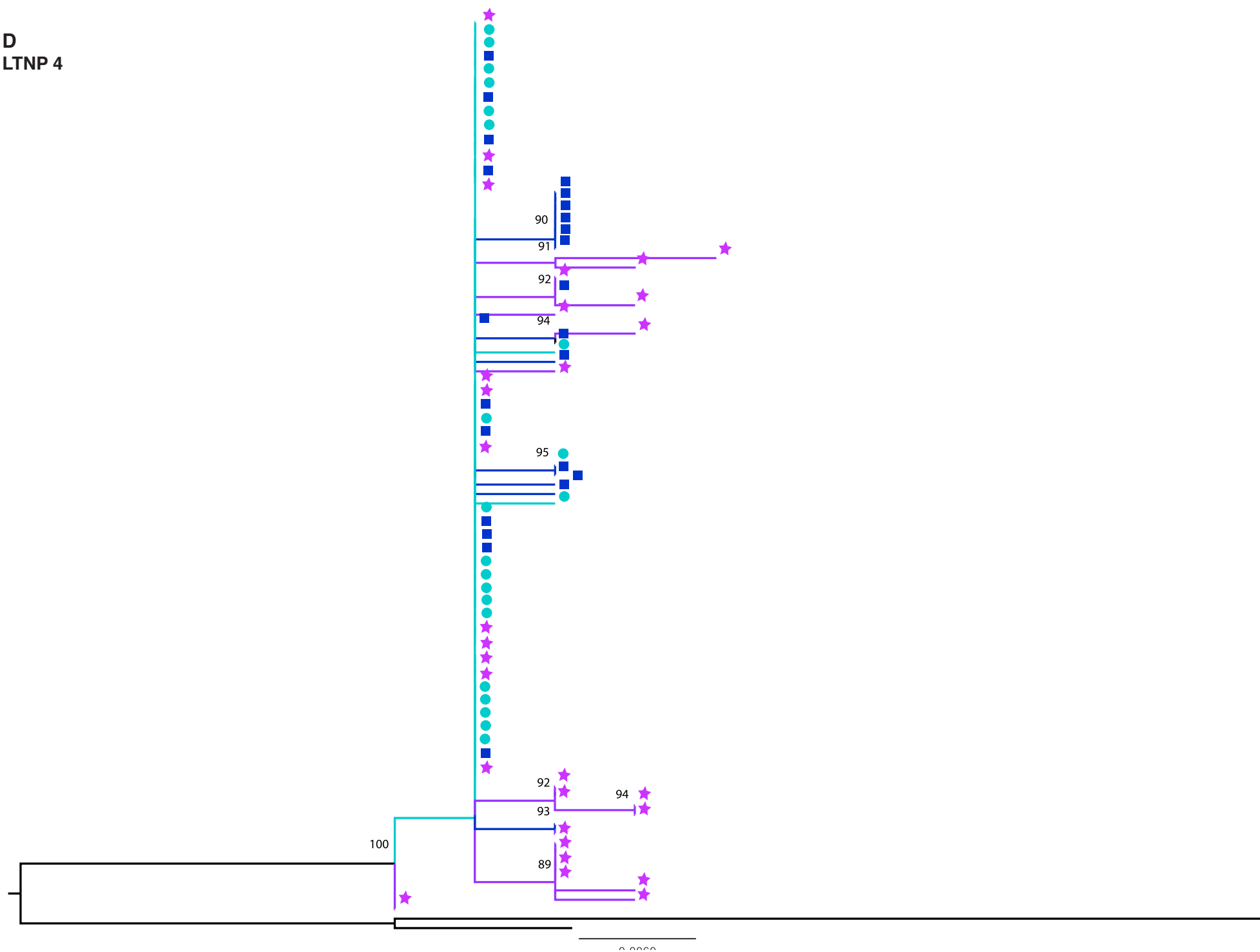

E  
LTNP 5

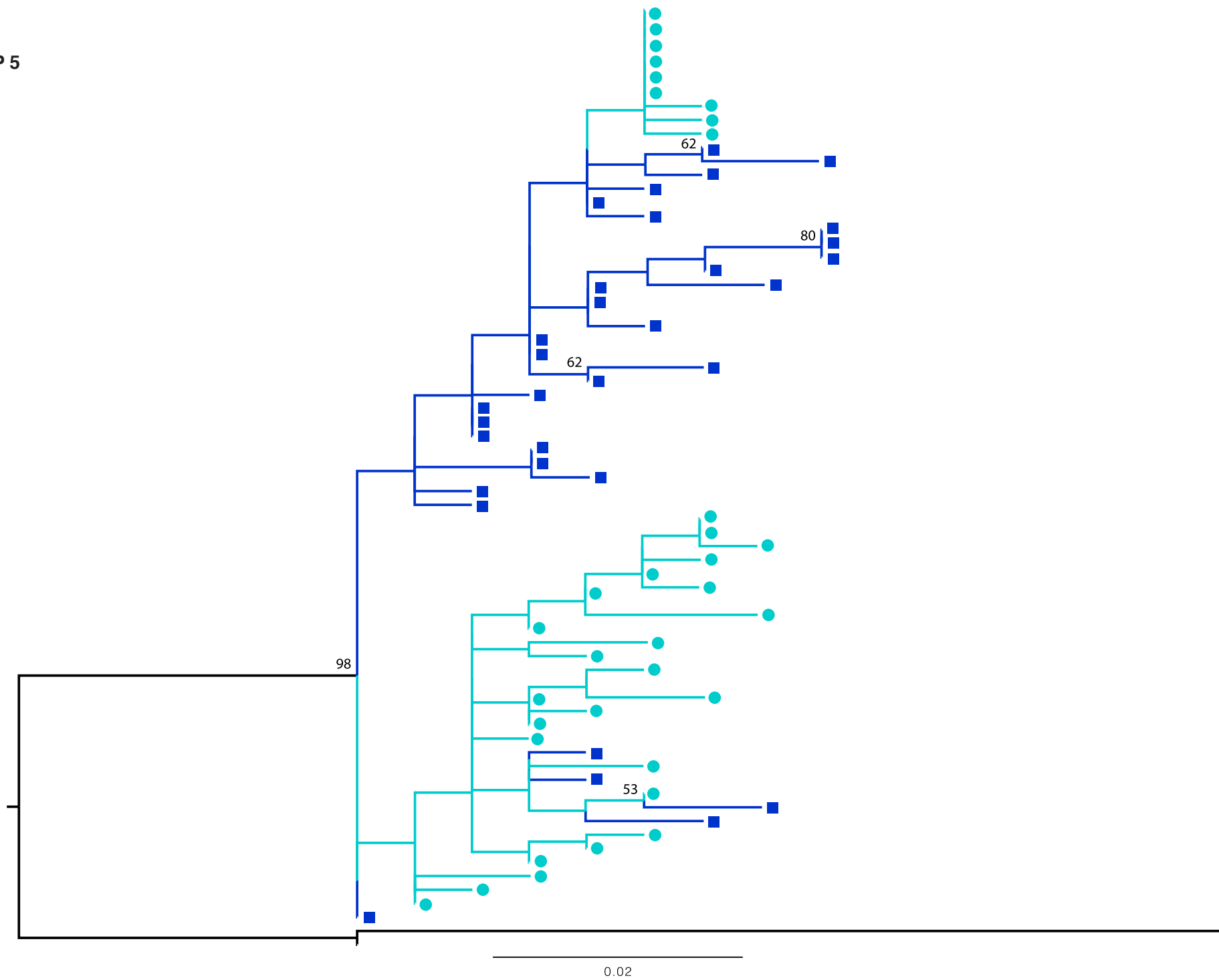

F  
NP 1

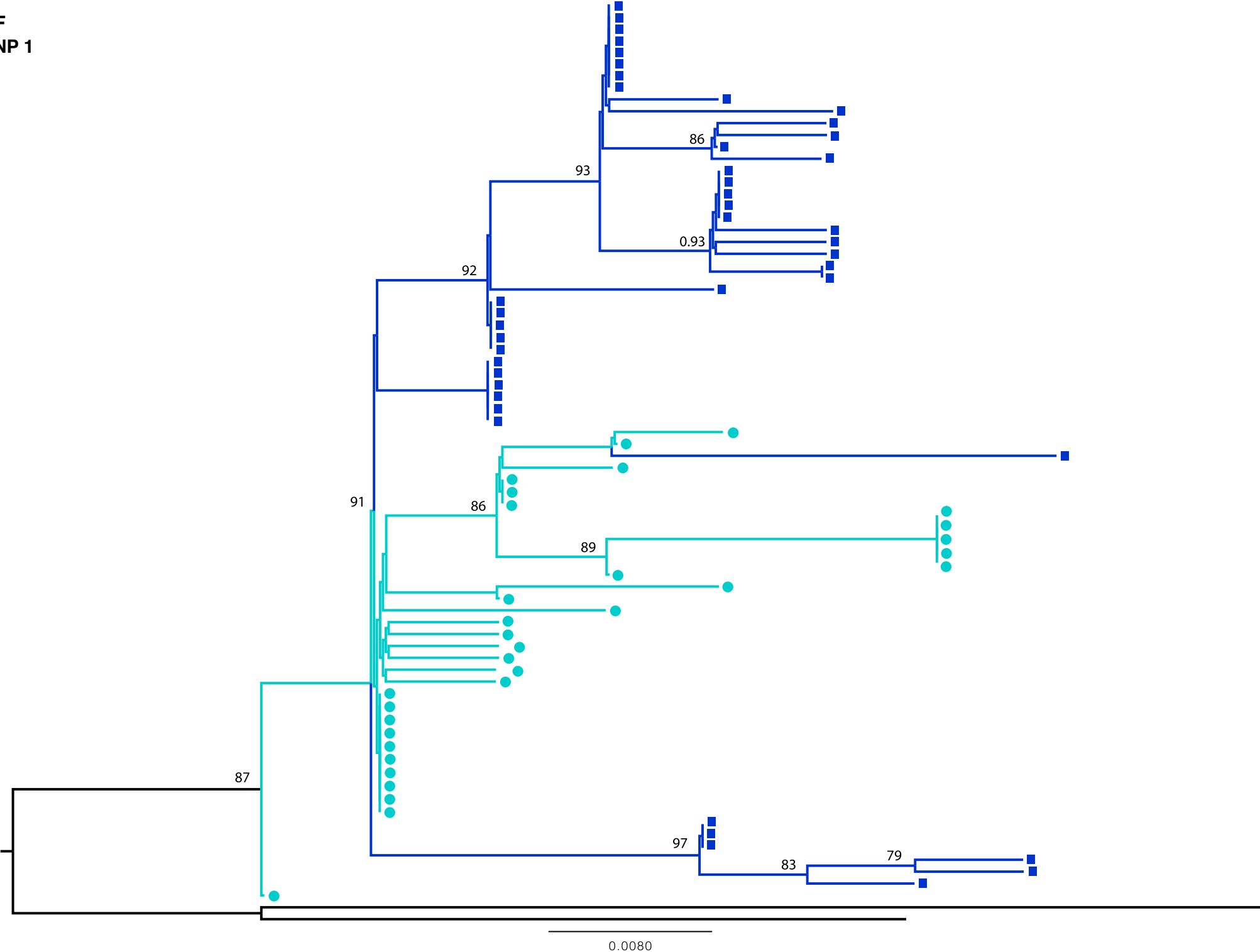

G  
NP 2

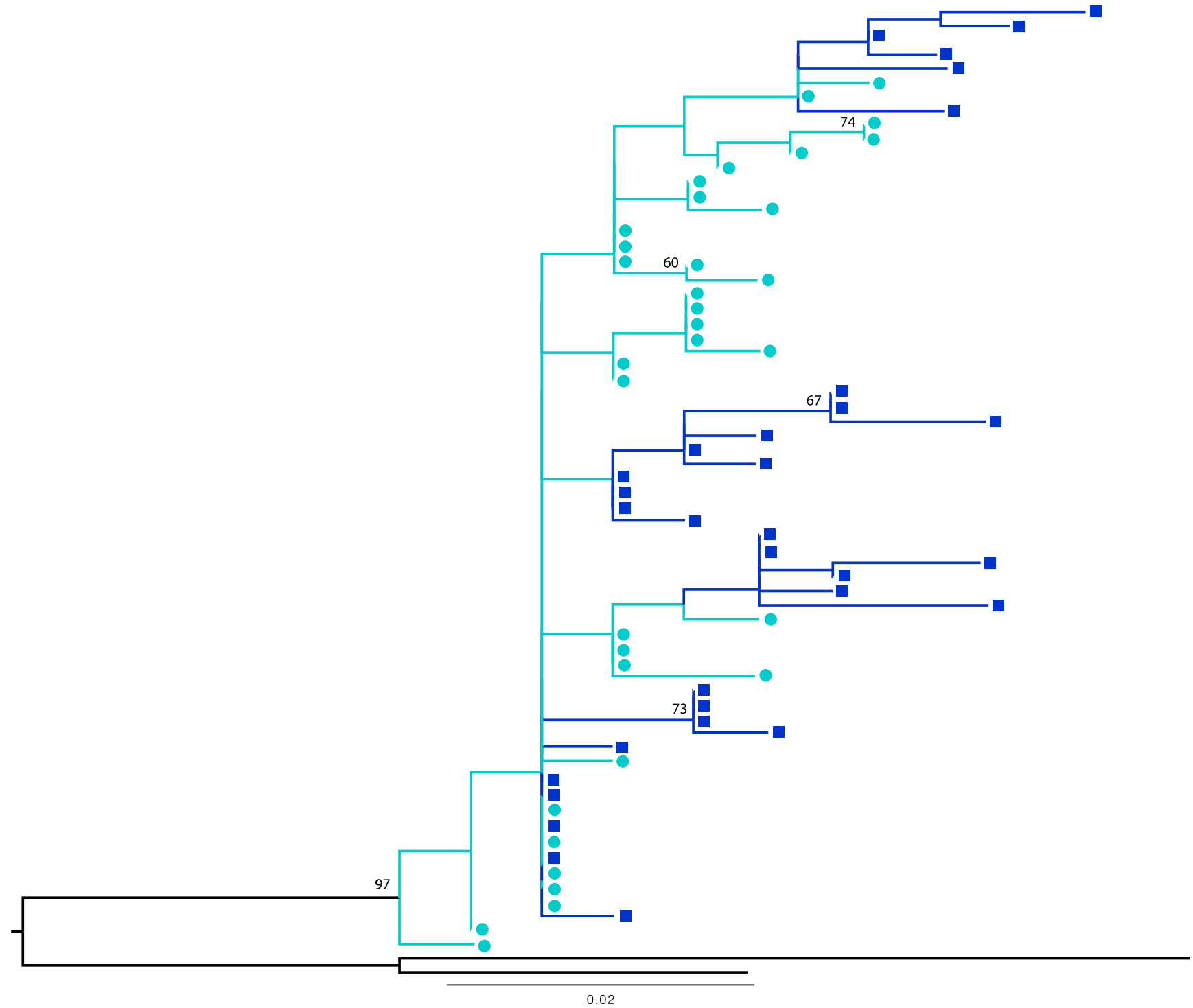

H  
NP 3

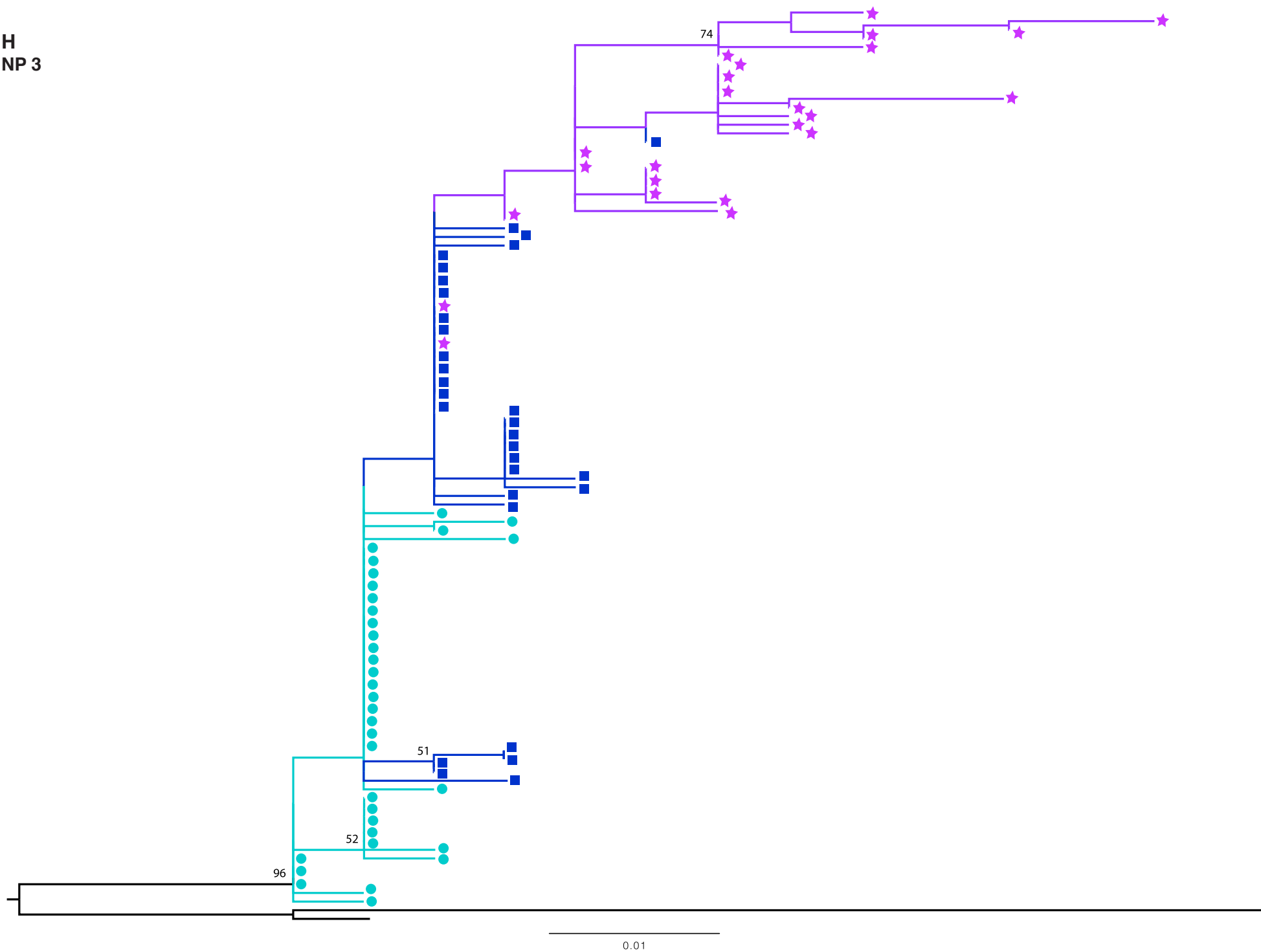

I  
NP 4

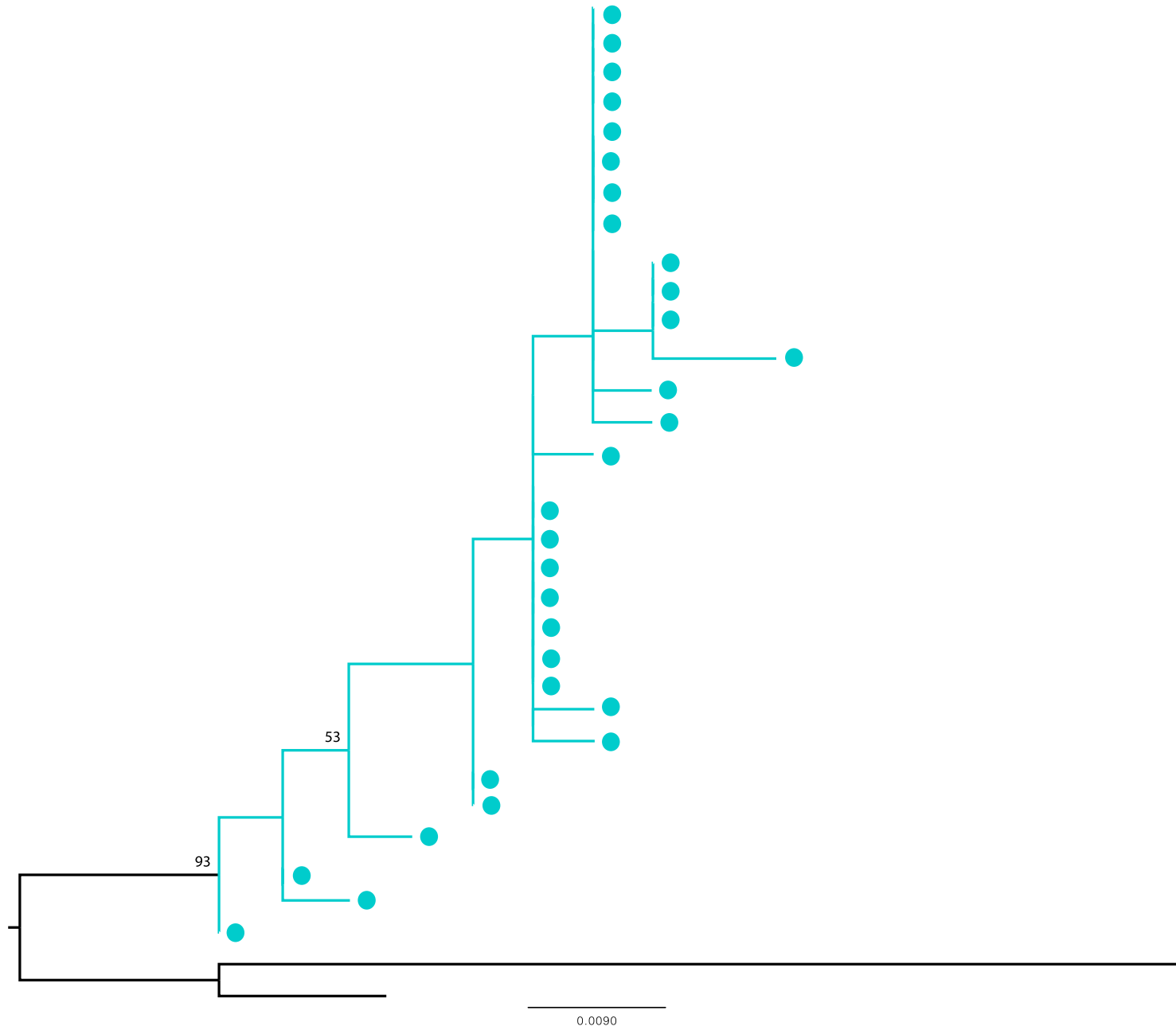

J  
RP 1

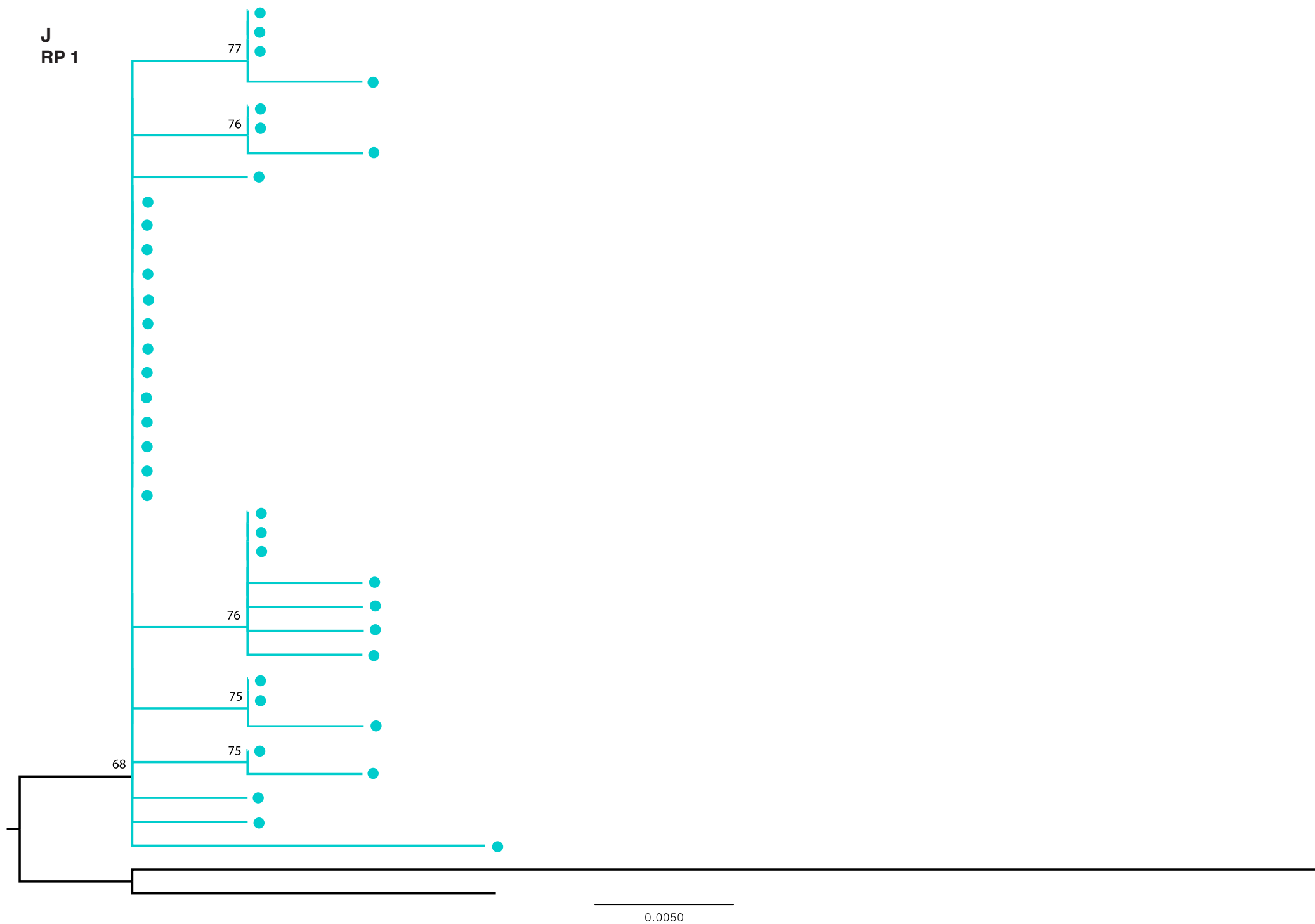

K  
RP 2

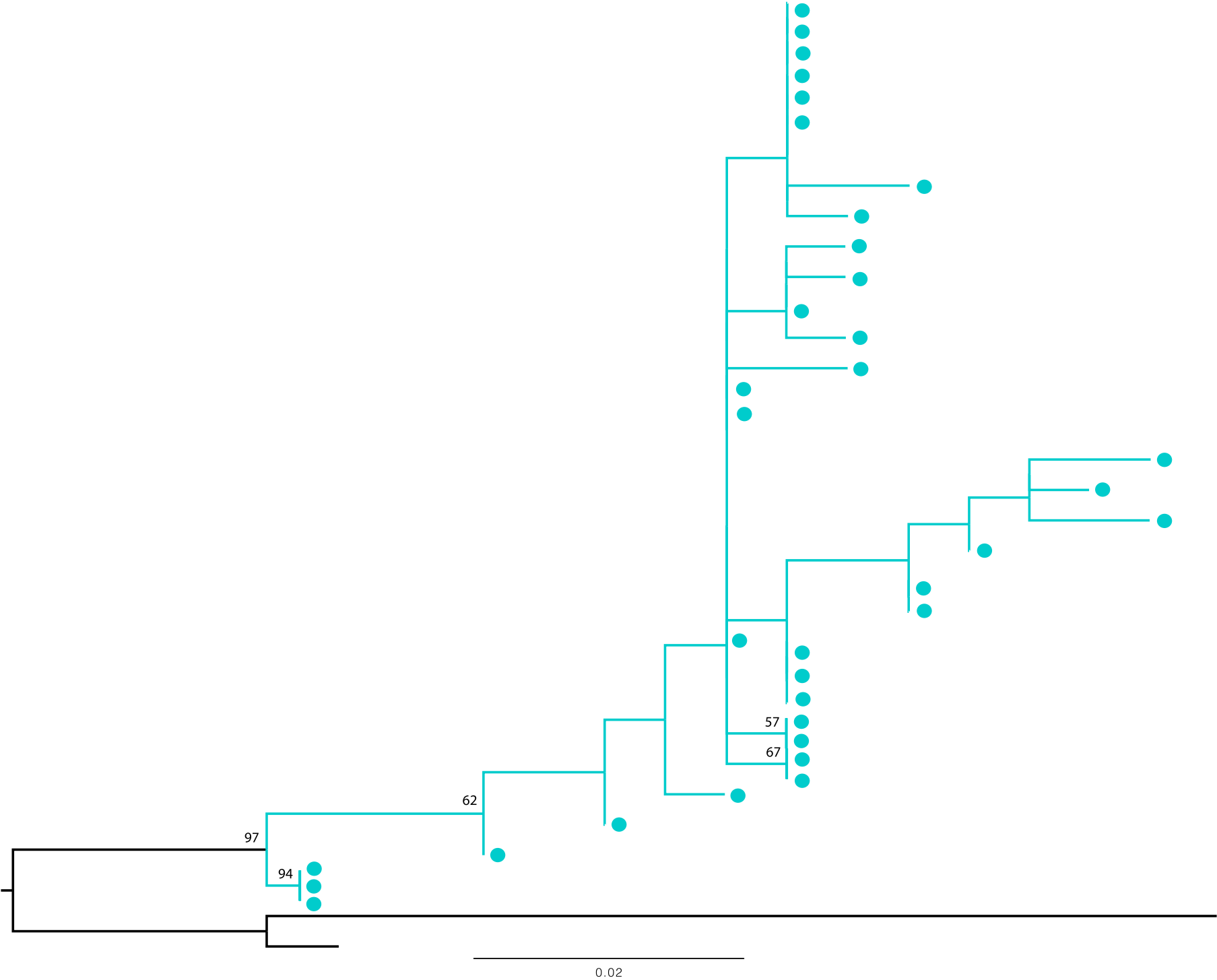

L  
RP 3

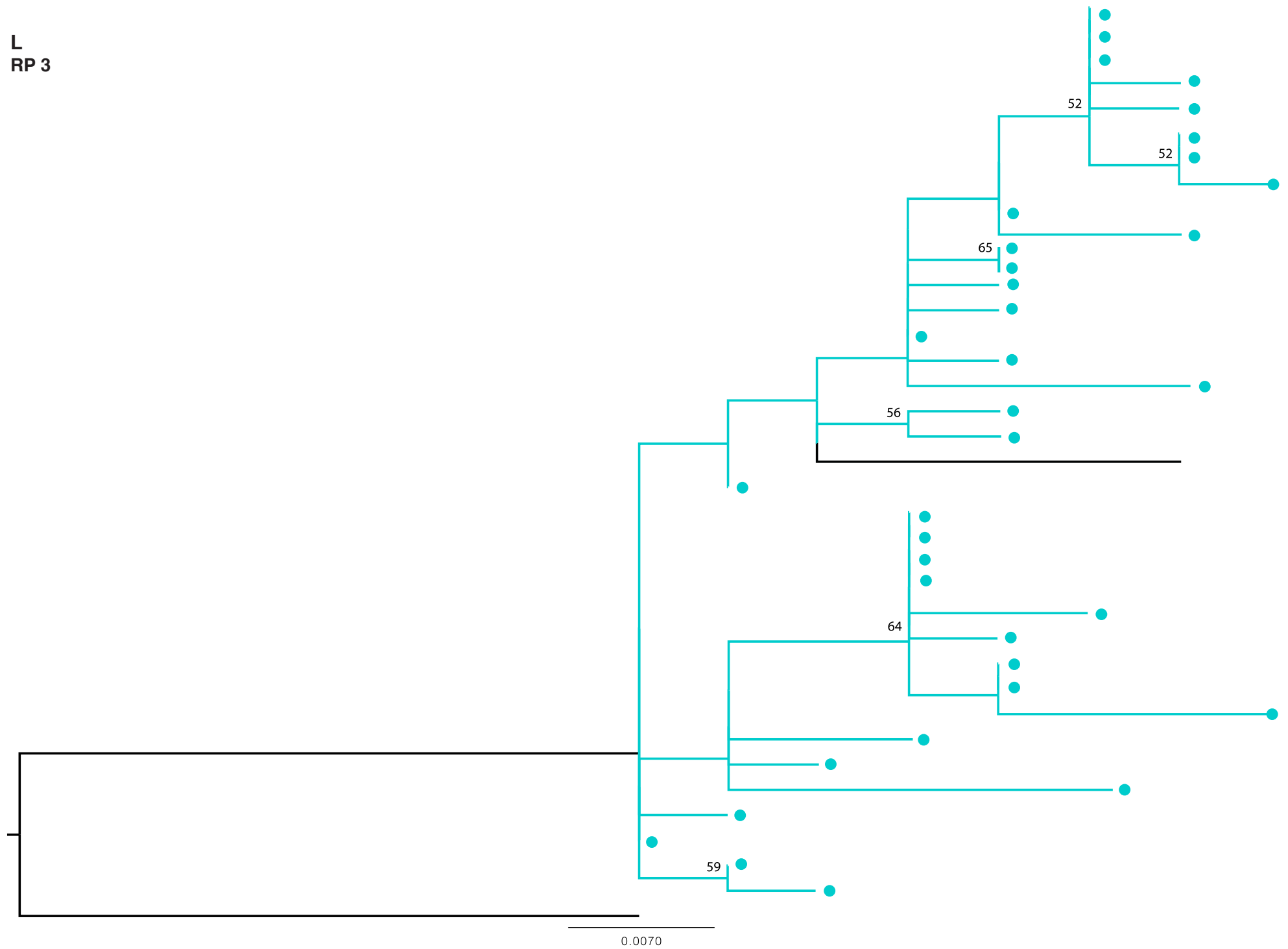

M  
RP 4

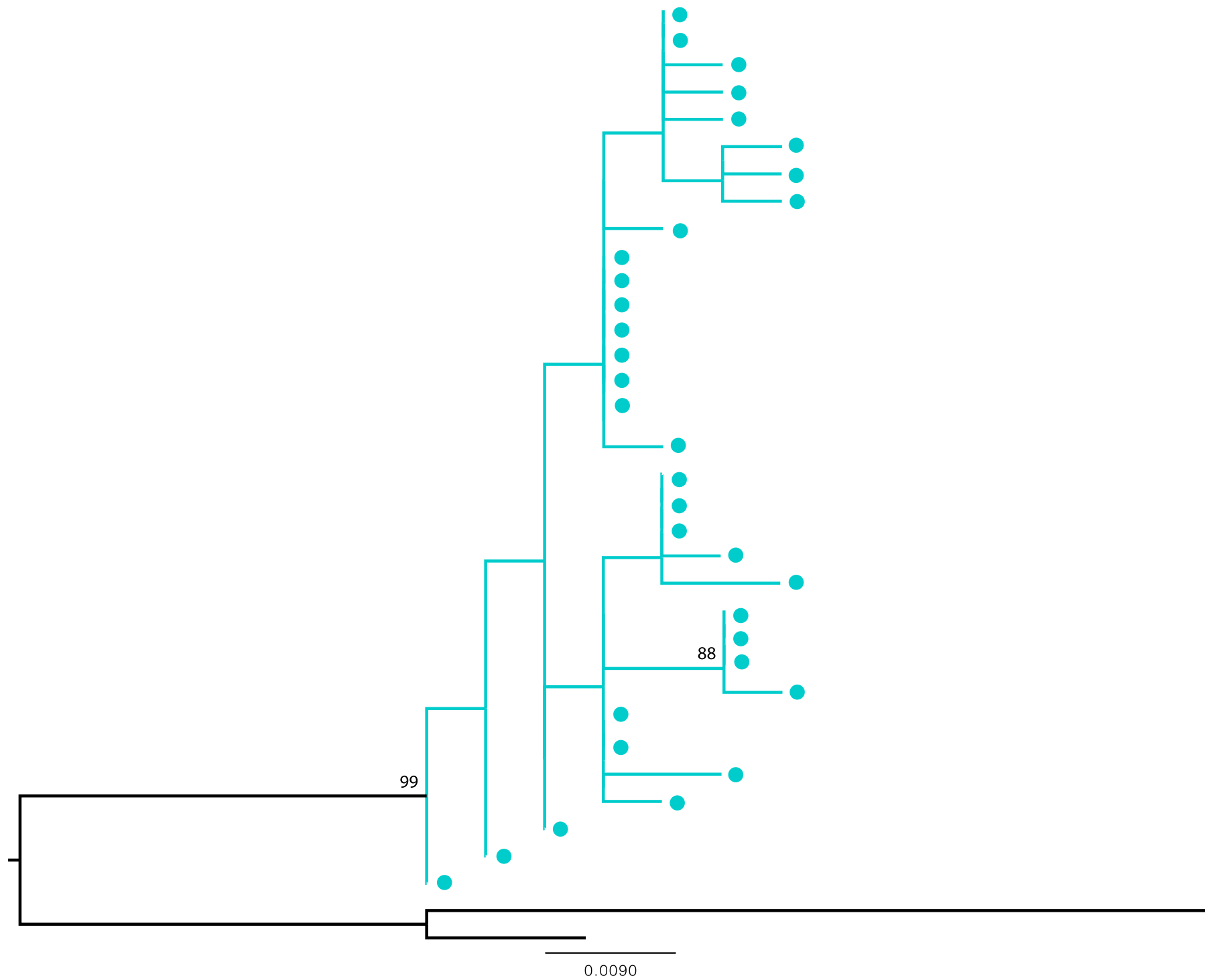

N  
RP 5

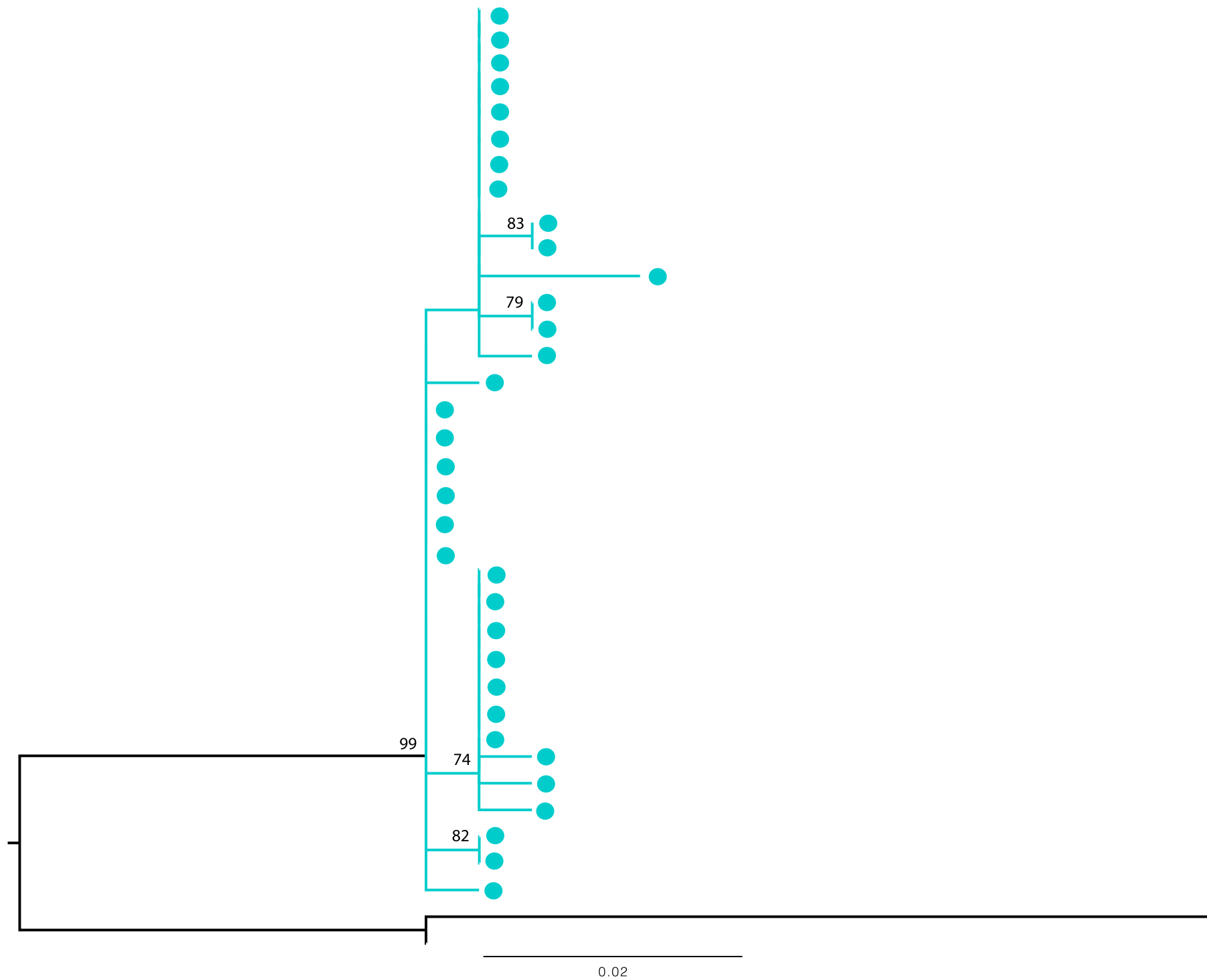

Supplement: Figure S1 — Phylogenetic trees of vpu sequences obtained from 14 HIV-1-infected individuals. Maximum likelihood phylogenies of vpu nucleotide sequences derived from 14 HIV-1-infected individuals. Bootstrap supports (% confidence) are shown at the base of the branch for each individual. Branch lengths indicate the number of nucleotide substitutions per site. The trees are rooted against NL4.3 and consensus B vpu sequences (black branches). Where multiple time points were available from one individual, earliest samples are depicted in turquoise circles; dark blue squares for intermediate time points; and purple stars for the later time point. (A) LTNP 1 (1.1, 4.4 and 10.4 yrs); (B) LTNP 2 (1.8 and 4.2 yrs); (C) LTNP 3 (1.2 and 4.5 yrs); (D) LTNP 4 (0, 1.1 and 3.5 yrs); (E) LTNP 5 (1.1 and 3.6 yrs); (F) NP 1 (1.6 and 3.5 yrs); (G) NP 2 (2 and 4.2 yrs); (H) NP 3 (0, 1.6 and 3.9 yrs); (I) NP 4 (4.9 yrs); (J) RP 1 (1.2 yrs); (K) RP 2 (1.0 yrs); (L) RP 3 (2.0 yrs); (M) RP 4 (2.0 yrs); and (N) RP 5 (1.1 yrs). The phylogenies of LTNP 3 and NP 1 were reconstructed after exclusion from the alignment of polymorphic residues showing evidence of reversion to wild type over time (codon positions 37 and 22 for LTNP 3 and NP 1 respectively), causing the artefactual clustering of late sequences closer to the outgroups than to those from the earlier time points. (PDF) [file ppat.1003895.s001.pdf]

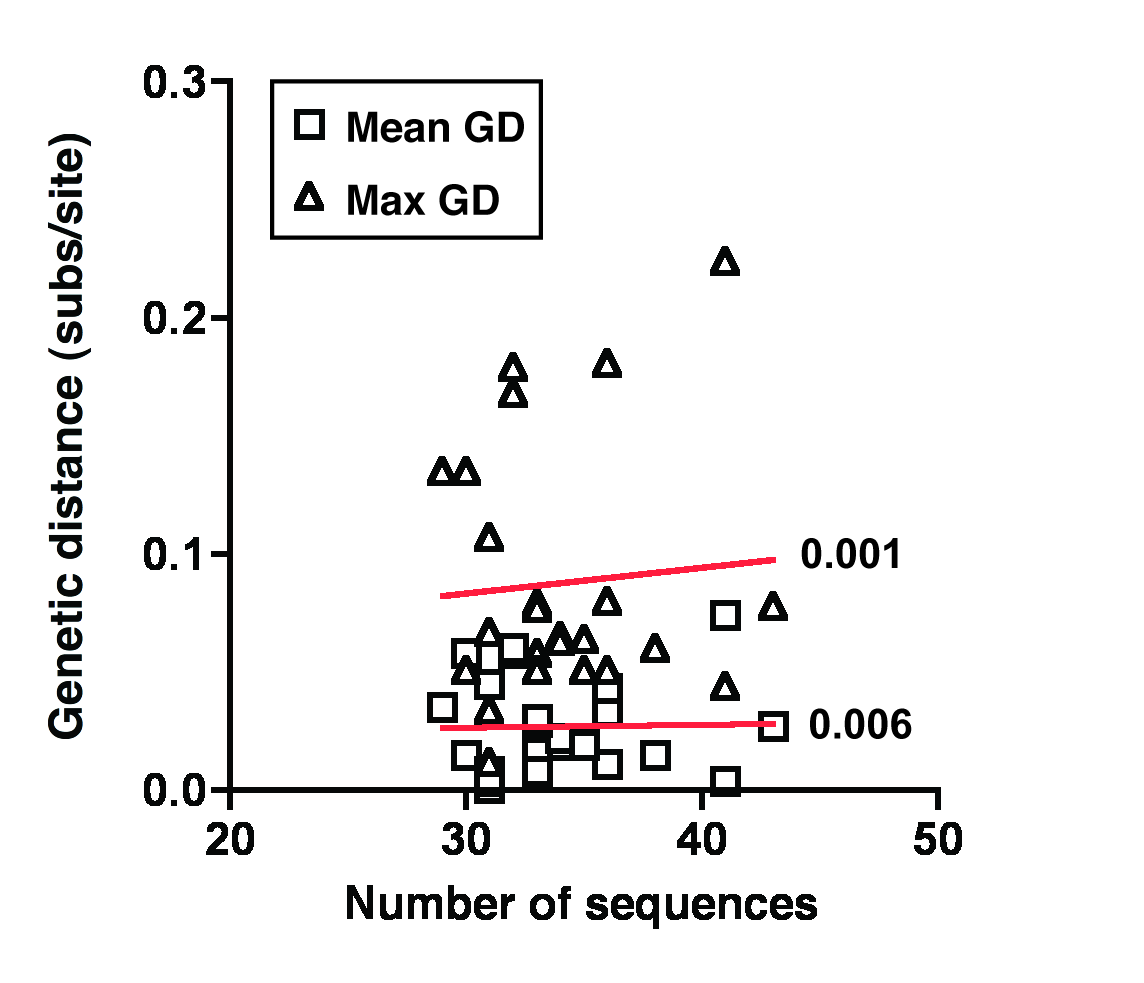

Supplement: Figure S2 — Genetic distance vs sequence number. Mean and maximum genetic distance values (GD; nucleotide substitutions per site) for each clinical sample used in this study were plotted against the total number of vpu sequences obtained per sample, as shown in Table 1 . R2 values are shown next to the slopes. A lack of correlation between the parameters is consistent with the vpu sampling number being sufficient to represent the viral population in peripheral blood at a given time point. (TIF) [file ppat.1003895.s002.tif]

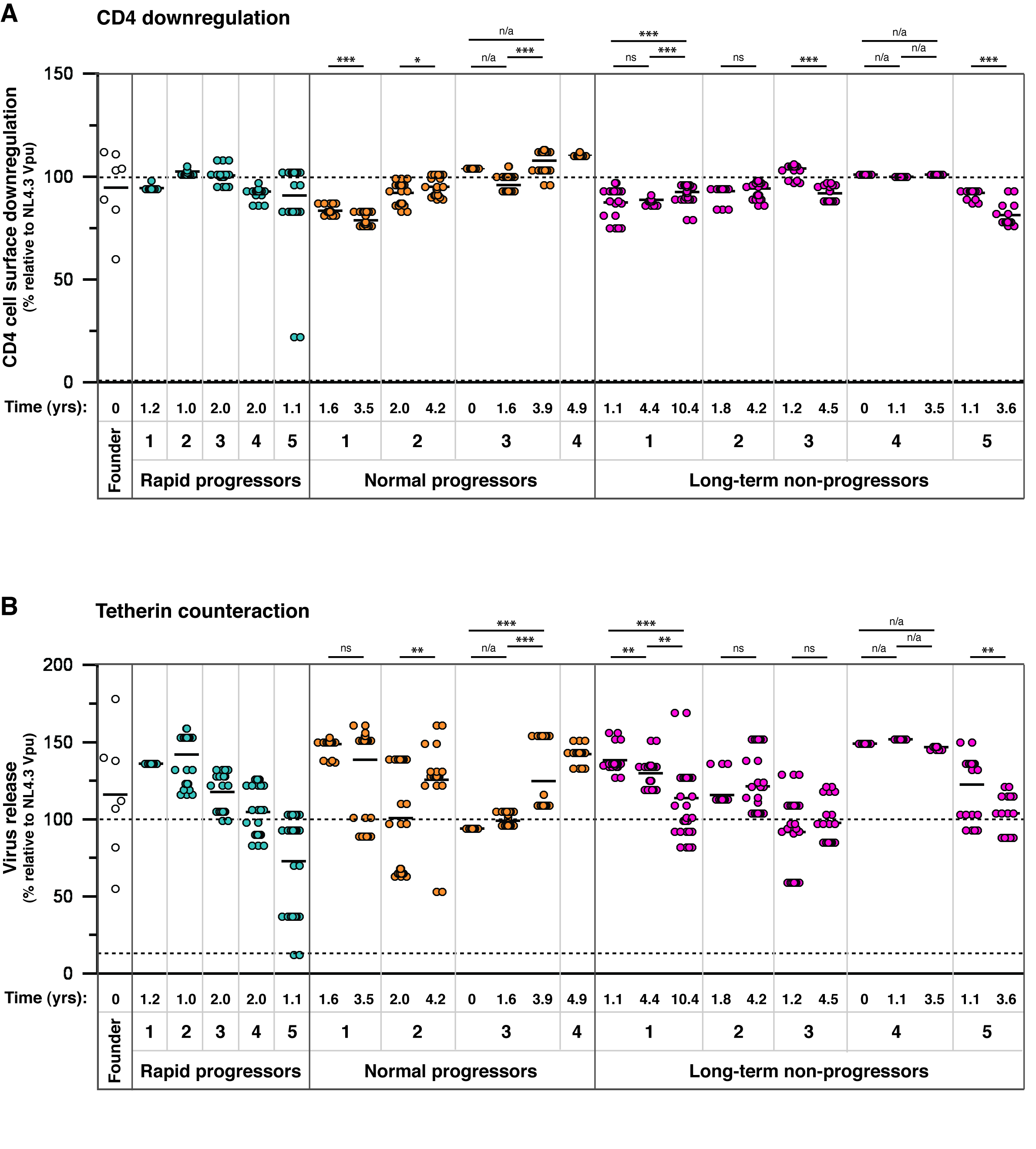

Supplement: Figure S3 — CD4 downregulation and tetherin counteraction of only multiple variants. Data from Figure 2 for CD4 downregulation (A) and tetherin counteraction (B) were re-plotted after the removal of all Vpus derived from a single genome to assess the impact of potentially transient variants on the outcome of the analyses. See legend from Figure 2 for details. Differences in function between time points were re-analysed for significance; in some cases, removal of single variants lead to all values for one time point being identical, and in these cases statistical analyses could not be applied (n/a; LTNP 3, LTNP 4). (TIF) [file ppat.1003895.s003.tif]

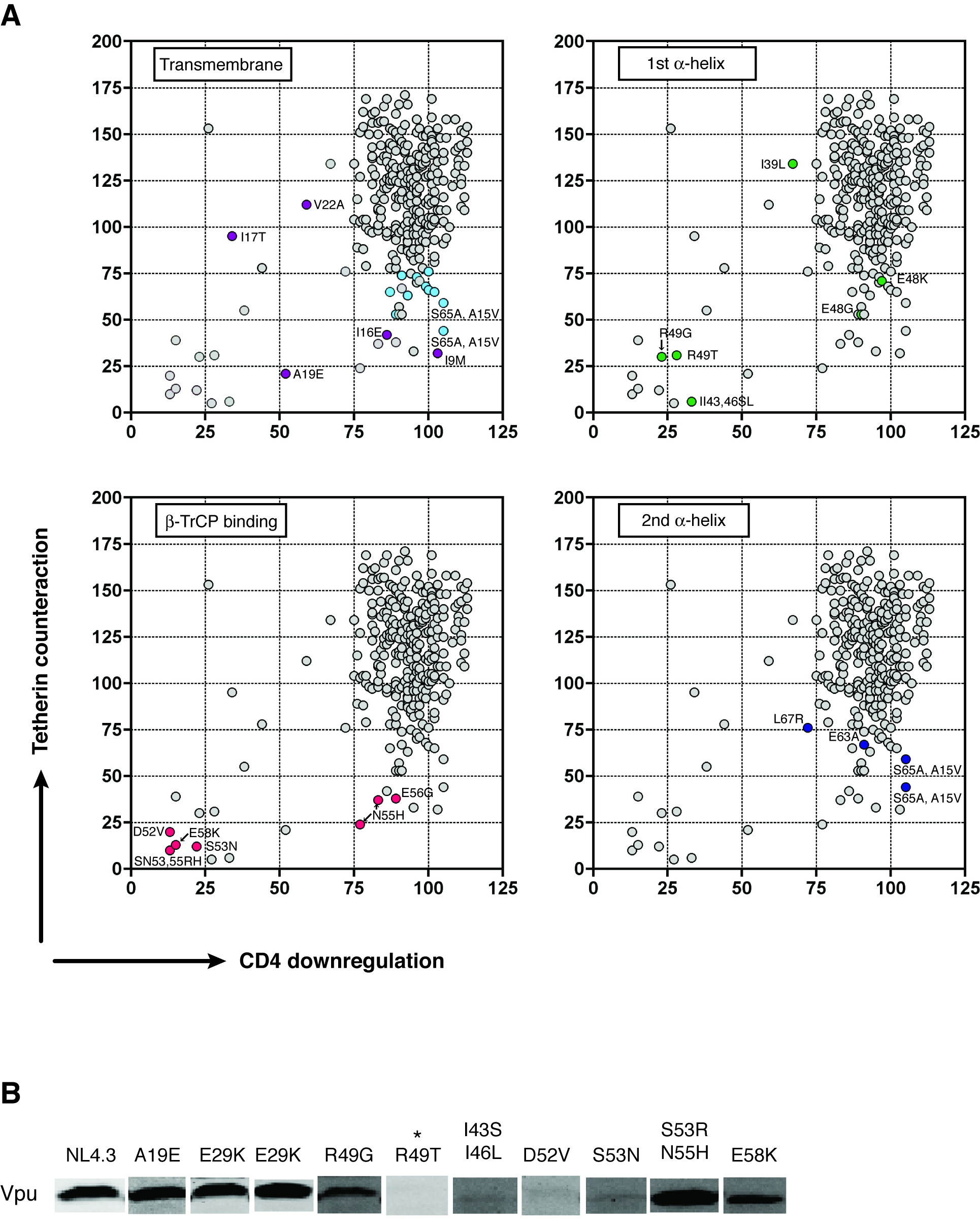

Supplement: Figure S4 — Detailed annotation of defective Vpu variants. (A) Defective and suboptimal Vpus categorised according to the location in which the mutations responsible for their defects occur, with the specific amino acid change indicated for each allele. (B) Vpus with defects in both functions were checked for expression by Western blot analysis. The only construct from which no Vpu expression could be detected is marked with an asterisk, but in this case closely-related fully functional Vpus could not be detected either, suggesting a lack of antibody-reactivity. (TIF) [file ppat.1003895.s004.tif]

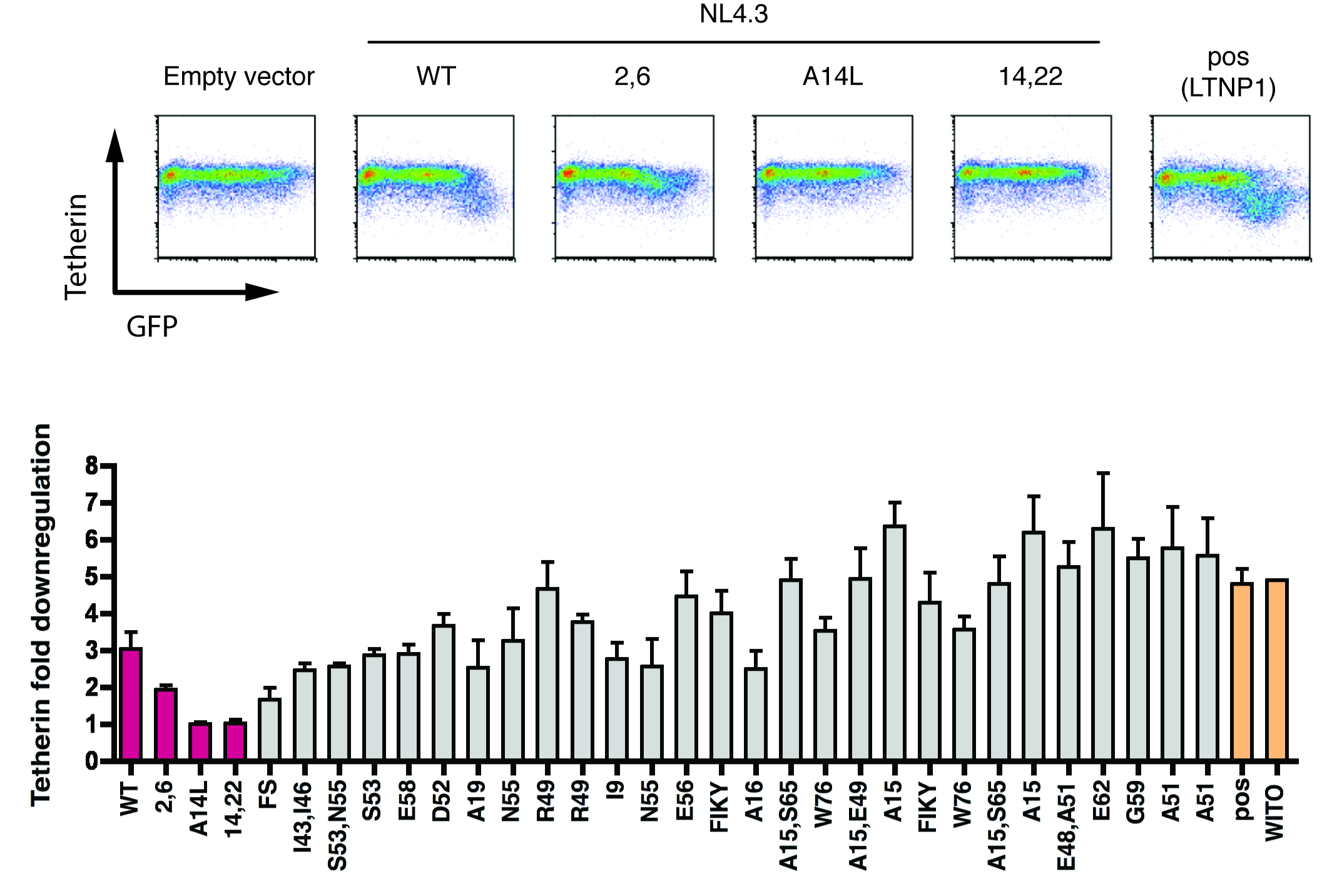

Supplement: Figure S5 — Ability of 30 patient-derived Vpus to downregulate cell surface tetherin levels. NL4.3 Vpu and three mutants thereof (S52,56A; A14L; and AW14,22LA), 28 patient-derived Vpus with various severe to minor defects in tetherin antagonism (see Figure 4 and Figure S4), a fully functional patient-derived Vpu (pos), and a founder virus-derived Vpu (WITO), were tested for their ability to downregulate tetherin cell-surface expression. TZMbl cells were transfected with 300 ng pCRVI-Vpu or EV and 300 ng pCR3.1-eGFP. 48 hours later cell surface tetherin levels were determined by flow cytometry. Fold tetherin downregulation was determined by comparing median fluorescent intensities of tetherin in the presence and absence of Vpu. Top panel: examples of FACS plots for EV, NL4.3 wt, S52,56A, A14L and AW14,22LA, and positive control Vpu LTNP1v4_1_67. Bottom panel: fold reduction of tetherin cell surface expression. Error bars represent standard deviation from the mean of 3 independent experiments. (TIF) [file ppat.1003895.s005.tif]

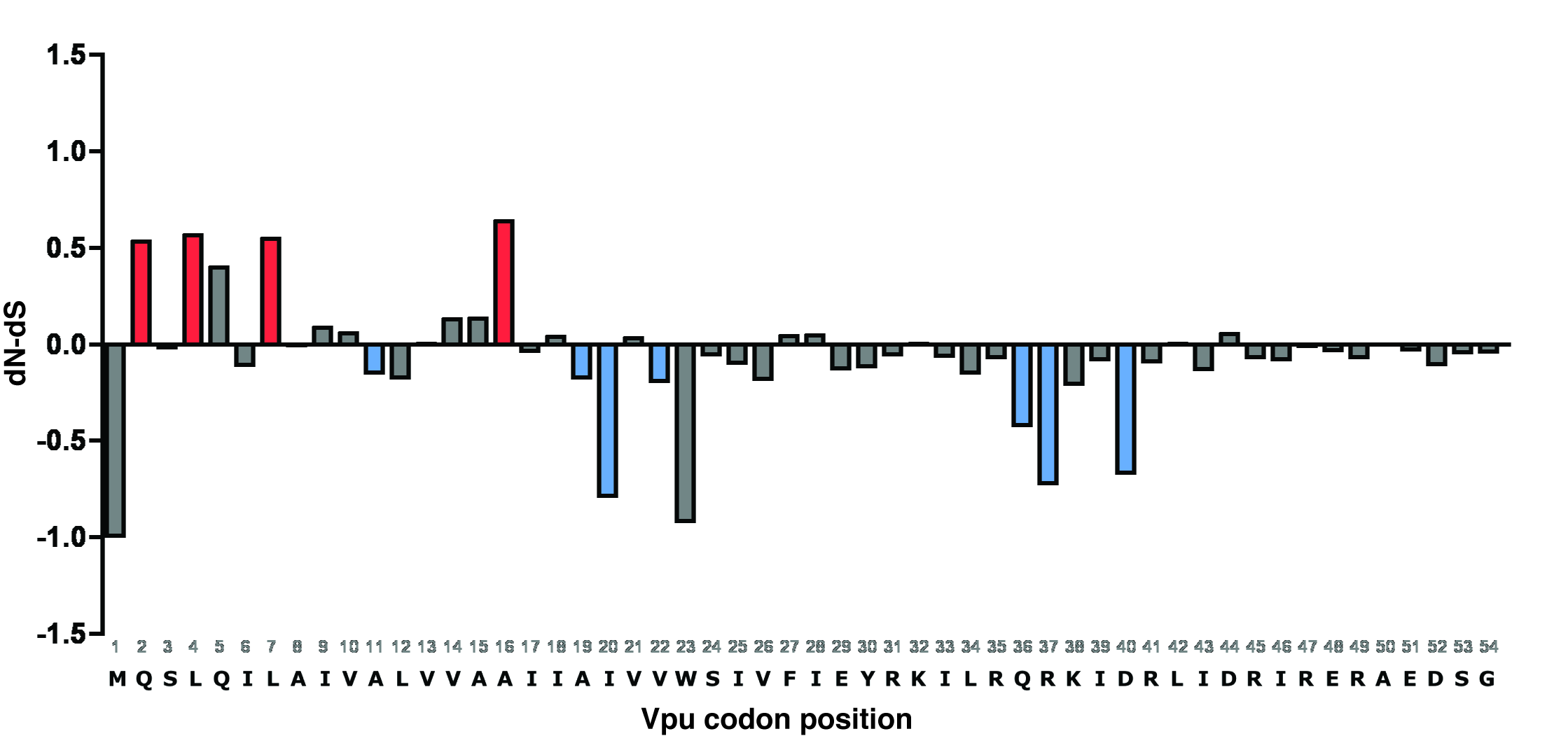

Supplement: Figure S6 — Positive/purifying selection acting on the vpu gene at the population level. All 851 sequences obtained were stripped of duplicates and analysed for codon-specific selective pressure at the population level. Three independent methods were used (SLAC, FEL and FUBAR) and FUBAR estimates of dN-dS are represented for each of the 81 codons across the vpu gene. Sites undergoing significant positive and negative selection are highlighted in red and blue respectively (posterior probablility >0.95). (TIF) [file ppat.1003895.s006.tif]
